# Supplementary material for: Early-stage lung adenocarcinoma affects DNA methylation and gene expression in adjacent tissues
Source: EMBO Rep. 2025 Nov 3;26(23):5931–58. doi: 10.1038/s44319-025-00612-4 (PMC12678790; doi:10.1038/s44319-025-00612-4)
Supplement: Supplementary file 1 — Appendix [file 44319_2025_612_MOESM1_ESM.pdf]

APPENDIX

Table of Contents

Appendix Figure S1 2
Appendix Figure S2 4
Appendix Figure S3 5
Appendix Figure S4 7
Appendix Figure S5 9
Appendix Figure S6 11
Appendix Figure S7 13
Appendix Figure S8 14
Appendix Figure S9 15
Appendix Figure S10 17
Appendix Methods 18

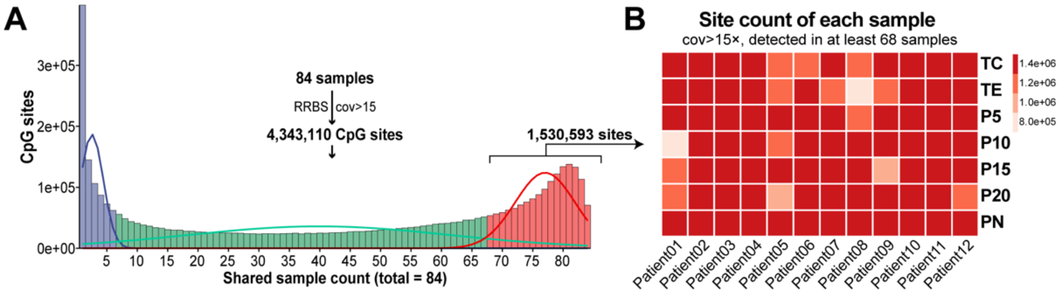

Appendix Figure S1. RRBS quality control and count of CpG sites in each sample.

(**A**) Frequency distribution of all detected CpG sites (n = 4,343,110) is presented. The threshold was determined at 68 based on the Gaussian mixture model analysis, which revealed three distributions at their intersection points. Consequently, 1,530,593 sites were retained for further analysis. (**B**) The number of detected CpG sites in each sample among the retained 1,530,593 CpG sites is illustrated.

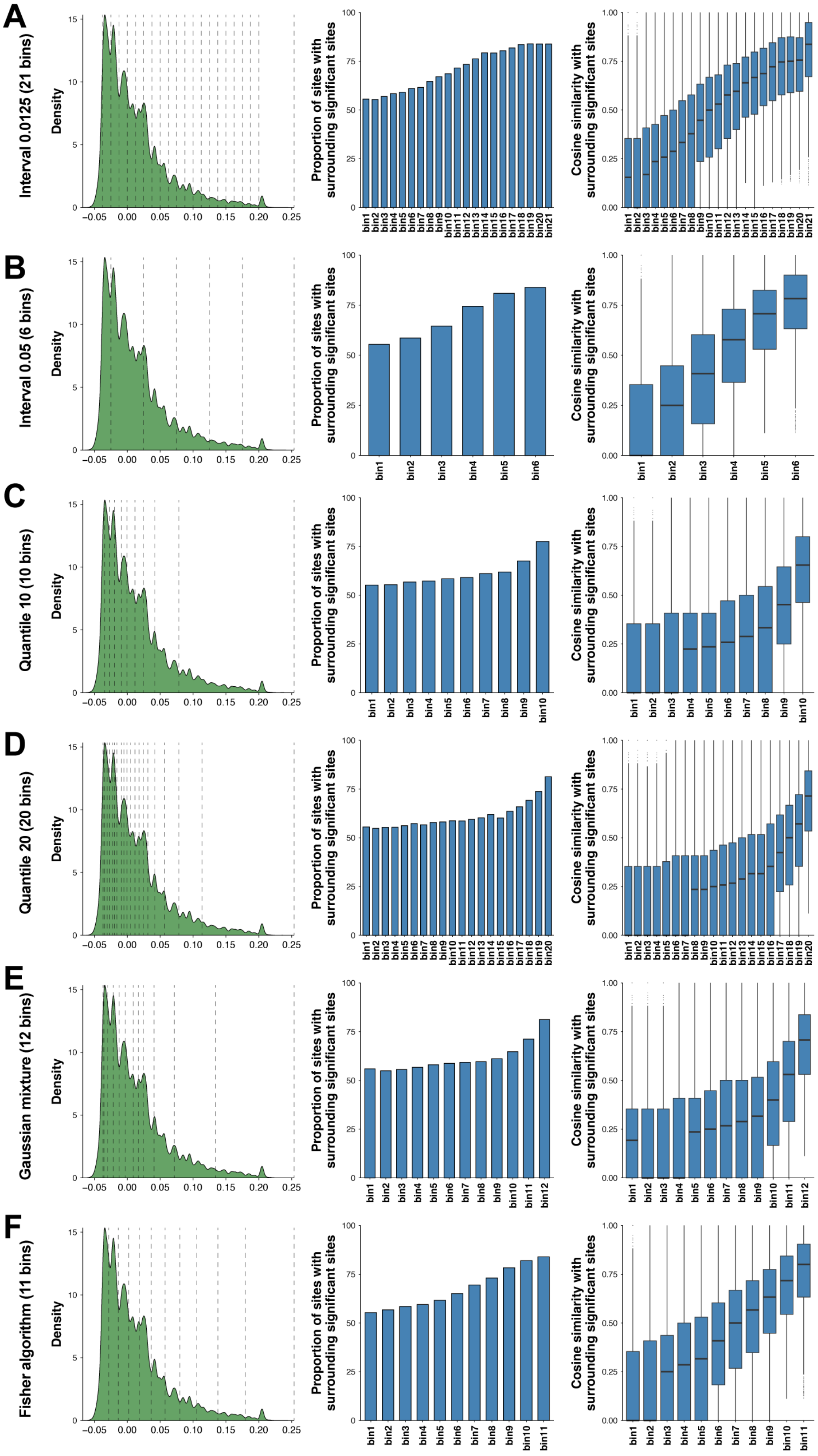

A
Interval 0.0125 (21 bins)
B
Interval 0.05 (6 bins)
C
Quantile 10 (10 bins)
D
Quantile 20 (20 bins)
E
Gaussian mixture (12 bins)
F
Fisher algorithm (11 bins)
Density
Proportion of sites with surrounding significant sites
Cosine similarity with surrounding significant sites

Appendix Figure S2. Robustness of the association between Moran's I and spatial ordering of sites across different binning strategies.

To evaluate the robustness of the observed correlation between Moran's *I* and spatial ordering of CpG sites, six binning strategies were applied: (**A**) fixed-width bins at 0.0125 intervals (21 bins), (**B**) fixed-width bins at 0.05 intervals (6 bins), (**C**) equal-sized quantile bins (10 bins), (**D**) quantile-based bins with finer resolution (20 bins), (**E**) bins generated by Gaussian mixture model (GMM) using the expectation-maximization (EM) algorithm (12 bins), and (**F**) bins defined by Fisher-Jenks natural breaks optimization (11 bins). For each strategy, we calculated the proportion of sites with at least one differentially methylated neighbor within ±30 nucleotides (middle panels) and the cosine similarity between each site and its neighbors (right panels). Across all binning methods, both metrics consistently increased with Moran's I, supporting the robustness of the observed ordering pattern. Detailed computational procedures and representative examples are provided in Fig. EV2.

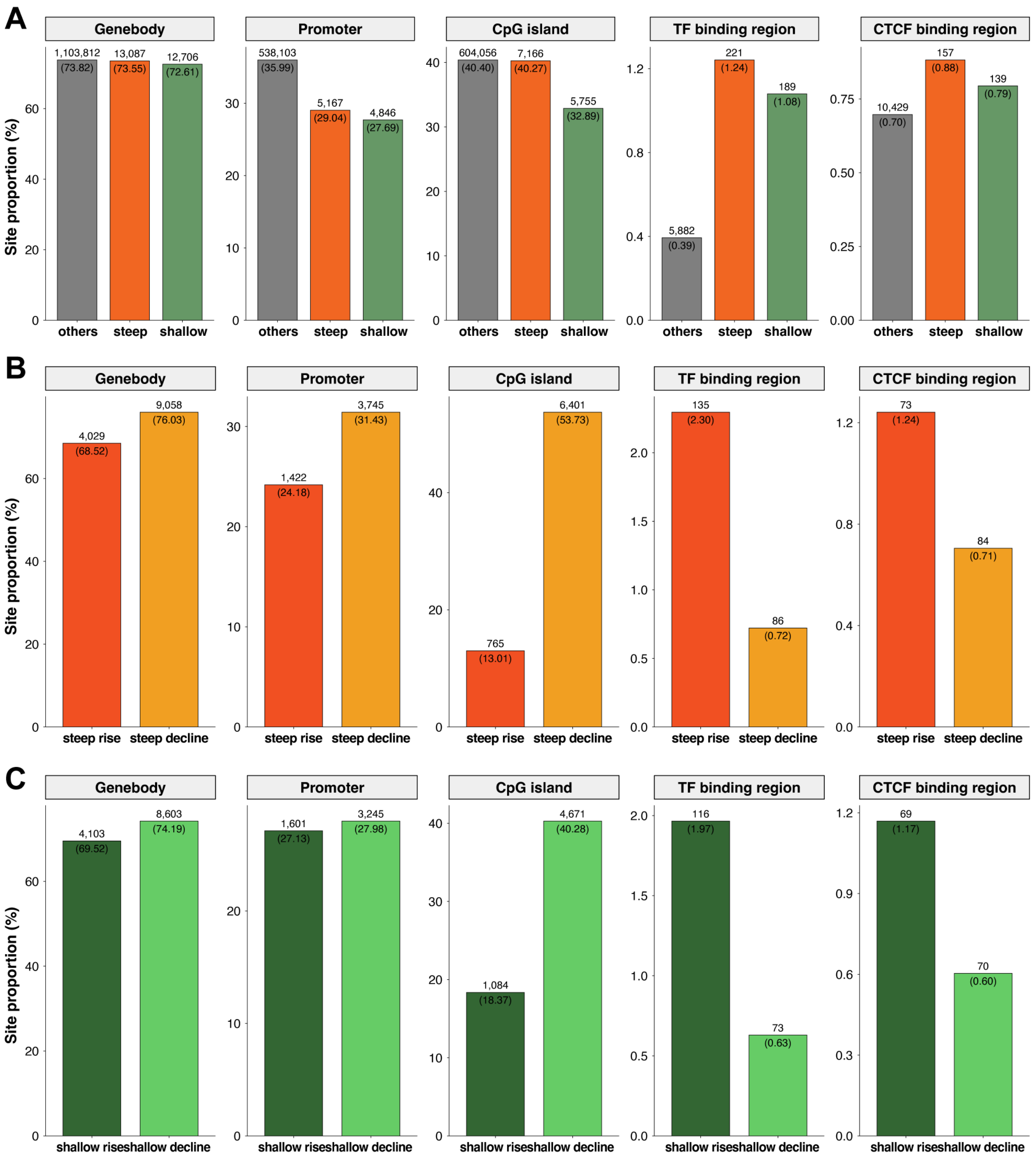

Appendix Figure S3. Genomic context distribution of CpG sites with steep and shallow methylation changes.

(**A**) Comparison of CpG sites with steep (n = 17,794, orange) and shallow (n = 17,498, green) methylation changes against all other detected CpG sites (n = 1,495,301, grey) across five genomic

features: gene bodies, promoters (2 kb upstream of TSS), CpG islands, transcription factor (TF) binding regions, and CTCF binding regions. (**B**) Feature distribution of steep rise and steep decline sites. (**C**) Feature distribution of shallow rise and shallow decline sites. Site counts and their corresponding proportions are labeled on each bar.

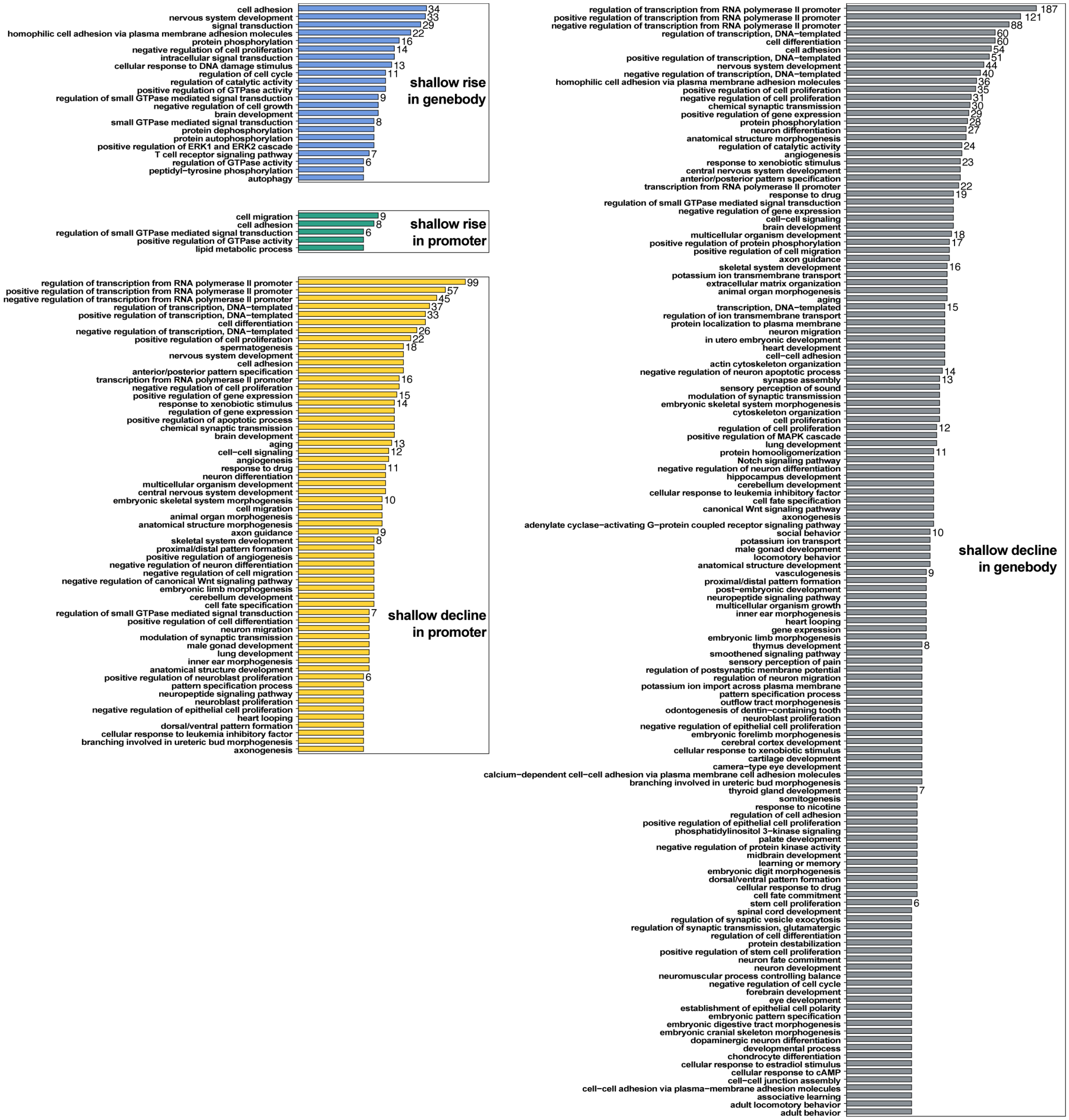

Appendix Figure S4. GO analysis of genes associated with methylation-changing regions.

GO analysis was conducted on genes containing only shallow- or mixed-changing regions, with a filter applied for gene count > 5 and P-value < 0.05. Bars represent relative gene counts. Genes with multiple changing regions were included only once per analysis for each changing type.

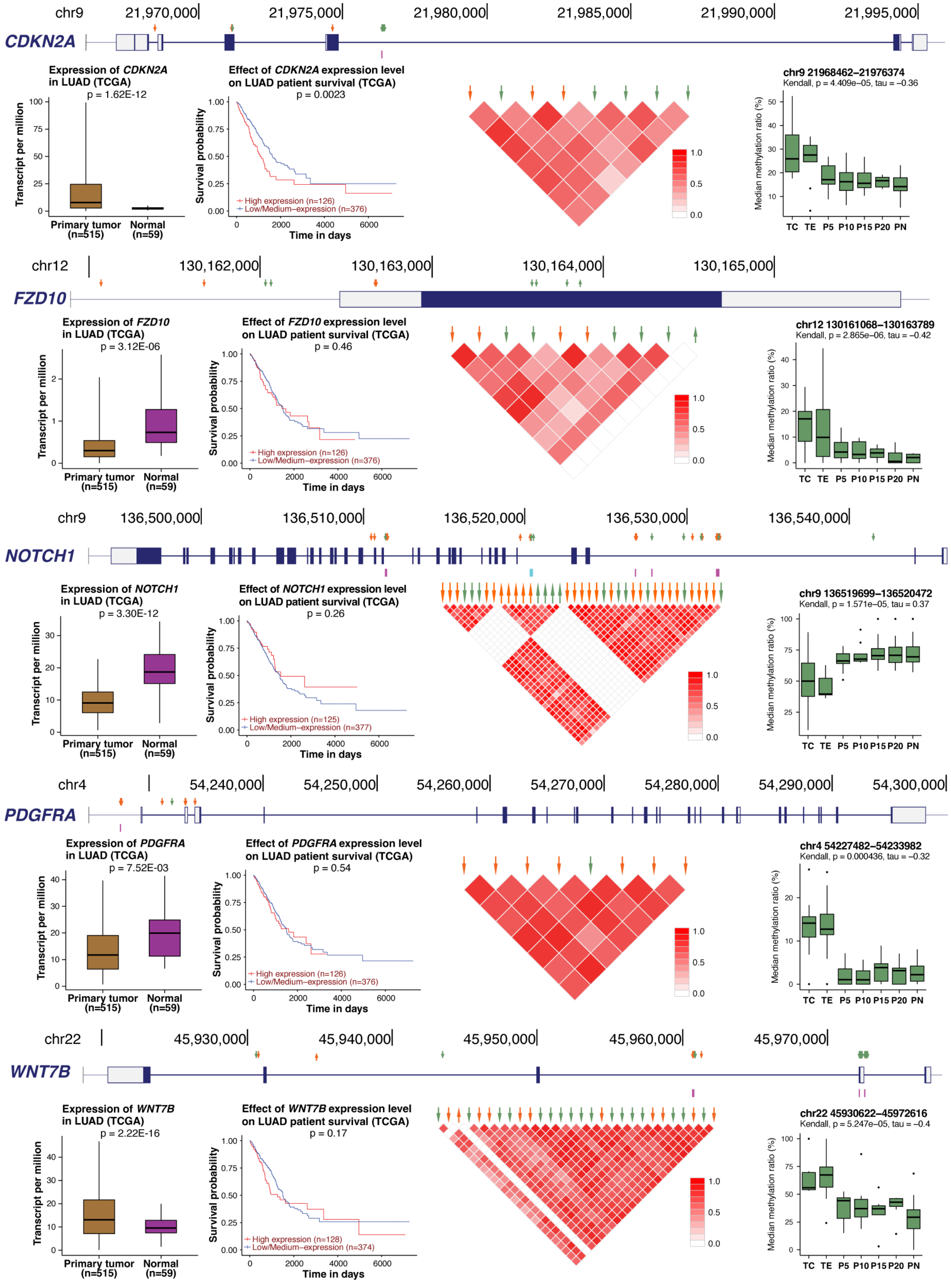

chr9
21,970,000
21,975,000
21,980,000
21,985,000
21,990,000
21,995,000
CDKN2A
Expression of CDKN2A in LUAD (TCGA)
p = 1.62E-12
Transcript per million
Primary tumor (n=515)
Normal (n=59)
Effect of CDKN2A expression level on LUAD patient survival (TCGA)
p = 0.0023
Survival probability
High expression (n=126)
Low/Medium-expression (n=376)
Time in days
chr9 21968462–21976374
Kendall, p = 4.409e-05, tau = −0.36
Median methylation ratio (%)
TC TE P5 P10 P15 P20 PN
chr12
130,162,000
130,163,000
130,164,000
130,165,000
FZD10
Expression of FZD10 in LUAD (TCGA)
p = 3.12E-06
Transcript per million
Primary tumor (n=515)
Normal (n=59)
Effect of FZD10 expression level on LUAD patient survival (TCGA)
p = 0.46
Survival probability
High expression (n=126)
Low/Medium-expression (n=376)
Time in days
chr12 130161068–130163789
Kendall, p = 2.865e-06, tau = −0.42
Median methylation ratio (%)
TC TE P5 P10 P15 P20 PN
chr9
136,500,000
136,510,000
136,520,000
136,530,000
136,540,000
NOTCH1
Expression of NOTCH1 in LUAD (TCGA)
p = 3.30E-12
Transcript per million
Primary tumor (n=515)
Normal (n=59)
Effect of NOTCH1 expression level on LUAD patient survival (TCGA)
p = 0.26
Survival probability
High expression (n=125)
Low/Medium-expression (n=377)
Time in days
chr9 136519699–136520472
Kendall, p = 1.571e-05, tau = 0.37
Median methylation ratio (%)
TC TE P5 P10 P15 P20 PN
chr4
54,240,000
54,250,000
54,260,000
54,270,000
54,280,000
54,290,000
54,300,000
PDGFRA
Expression of PDGFRA in LUAD (TCGA)
p = 7.52E-03
Transcript per million
Primary tumor (n=515)
Normal (n=59)
Effect of PDGFRA expression level on LUAD patient survival (TCGA)
p = 0.54
Survival probability
High expression (n=126)
Low/Medium-expression (n=376)
Time in days
chr4 54227482–54233982
Kendall, p = 0.000436, tau = −0.32
Median methylation ratio (%)
TC TE P5 P10 P15 P20 PN
chr22
45,930,000
45,940,000
45,950,000
45,960,000
45,970,000
WNT7B
Expression of WNT7B in LUAD (TCGA)
p = 2.22E-16
Transcript per million
Primary tumor (n=515)
Normal (n=59)
Effect of WNT7B expression level on LUAD patient survival (TCGA)
p = 0.17
Survival probability
High expression (n=128)
Low/Medium-expression (n=374)
Time in days
chr22 45930622–45972616
Kendall, p = 5.247e-05, tau = −0.4
Median methylation ratio (%)
TC TE P5 P10 P15 P20 PN

Appendix Figure S5. Methylation-changing CpG sites and transcriptional features of five target genes.

Genomic distributions of methylation-changing CpG sites are shown first. Gene expression levels of the five genes in primary LUAD and normal tissues based on TCGA data. Kaplan–Meier survival curves of TCGA-LUAD patients stratified by expression levels of each gene. Cosine similarity of CpG sites on each gene. Median methylation levels across seven spatially resolved tissue locations (TC, TE, P5, P10, P15, P20, PN) for each gene, were calculated from CpG sites located in genebody and promoter regions.

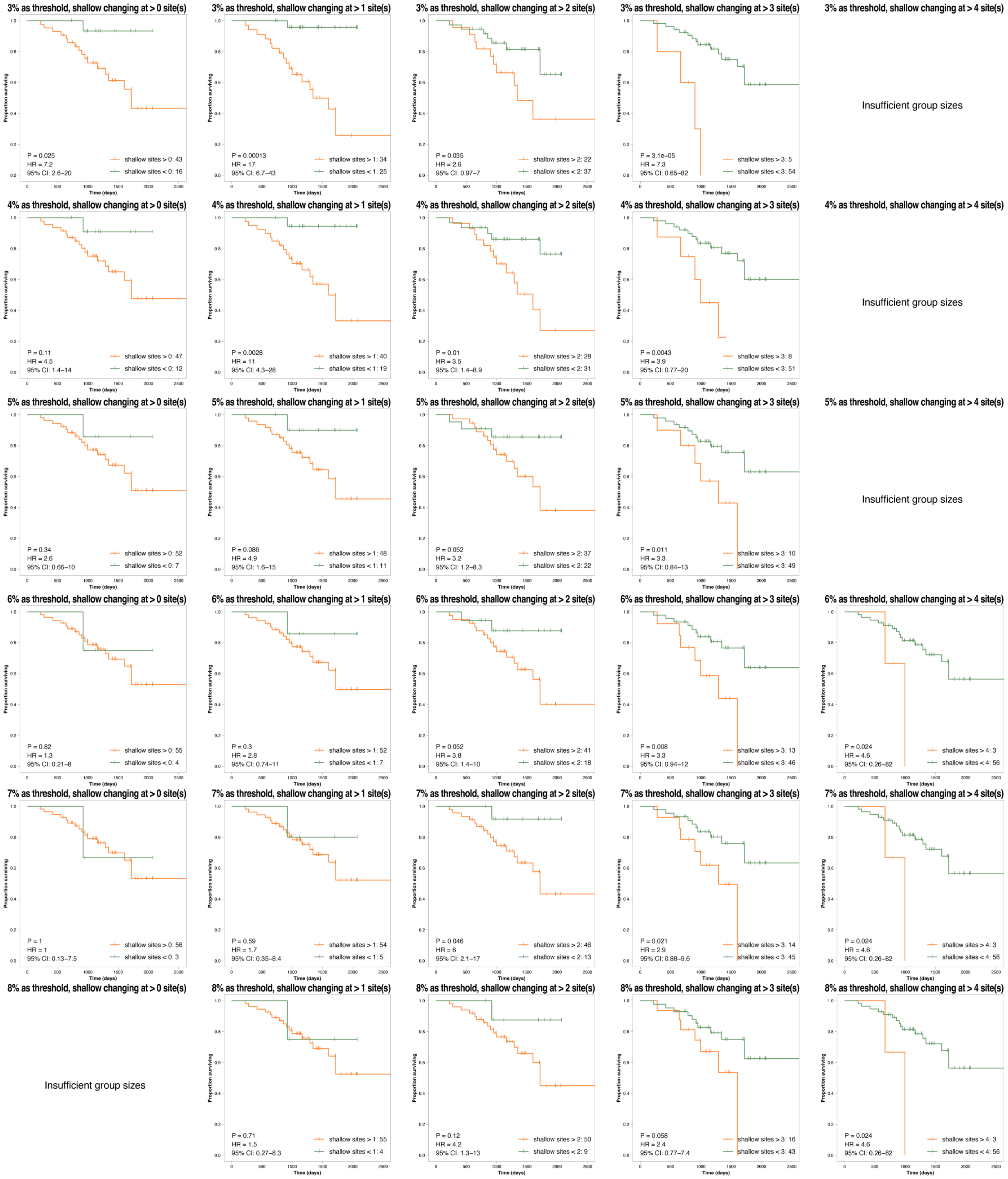

3% as threshold, shallow changing at > 0 site(s)
P = 0.025
HR = 7.2
95% CI: 2.6–20
shallow sites > 0: 43
shallow sites < 0: 16
3% as threshold, shallow changing at > 1 site(s)
P = 0.00013
HR = 17
95% CI: 6.7–43
shallow sites > 1: 34
shallow sites < 1: 25
3% as threshold, shallow changing at > 2 site(s)
P = 0.035
HR = 2.6
95% CI: 0.97–7
shallow sites > 2: 22
shallow sites < 2: 37
3% as threshold, shallow changing at > 3 site(s)
P = 3.1e−05
HR = 7.3
95% CI: 0.65–82
shallow sites > 3: 5
shallow sites < 3: 54
3% as threshold, shallow changing at > 4 site(s)
Insufficient group sizes
4% as threshold, shallow changing at > 0 site(s)
P = 0.11
HR = 4.5
95% CI: 1.4–14
shallow sites > 0: 47
shallow sites < 0: 12
4% as threshold, shallow changing at > 1 site(s)
P = 0.0028
HR = 11
95% CI: 4.3–28
shallow sites > 1: 40
shallow sites < 1: 19
4% as threshold, shallow changing at > 2 site(s)
P = 0.01
HR = 3.5
95% CI: 1.4–8.9
shallow sites > 2: 28
shallow sites < 2: 31
4% as threshold, shallow changing at > 3 site(s)
P = 0.0043
HR = 3.9
95% CI: 0.77–20
shallow sites > 3: 8
shallow sites < 3: 51
4% as threshold, shallow changing at > 4 site(s)
Insufficient group sizes
5% as threshold, shallow changing at > 0 site(s)
P = 0.34
HR = 2.6
95% CI: 0.66–10
shallow sites > 0: 52
shallow sites < 0: 7
5% as threshold, shallow changing at > 1 site(s)
P = 0.086
HR = 4.9
95% CI: 1.6–15
shallow sites > 1: 48
shallow sites < 1: 11
5% as threshold, shallow changing at > 2 site(s)
P = 0.052
HR = 3.2
95% CI: 1.2–8.3
shallow sites > 2: 37
shallow sites < 2: 22
5% as threshold, shallow changing at > 3 site(s)
P = 0.011
HR = 3.3
95% CI: 0.84–13
shallow sites > 3: 10
shallow sites < 3: 49
5% as threshold, shallow changing at > 4 site(s)
Insufficient group sizes
6% as threshold, shallow changing at > 0 site(s)
P = 0.82
HR = 1.3
95% CI: 0.21–8
shallow sites > 0: 55
shallow sites < 0: 4
6% as threshold, shallow changing at > 1 site(s)
P = 0.3
HR = 2.8
95% CI: 0.74–11
shallow sites > 1: 52
shallow sites < 1: 7
6% as threshold, shallow changing at > 2 site(s)
P = 0.052
HR = 3.8
95% CI: 1.4–10
shallow sites > 2: 41
shallow sites < 2: 18
6% as threshold, shallow changing at > 3 site(s)
P = 0.008
HR = 3.3
95% CI: 0.94–12
shallow sites > 3: 13
shallow sites < 3: 46
6% as threshold, shallow changing at > 4 site(s)
P = 0.024
HR = 4.6
95% CI: 0.26–82
shallow sites > 4: 3
shallow sites < 4: 56
7% as threshold, shallow changing at > 0 site(s)
P = 1
HR = 1
95% CI: 0.13–7.5
shallow sites > 0: 56
shallow sites < 0: 3
7% as threshold, shallow changing at > 1 site(s)
P = 0.59
HR = 1.7
95% CI: 0.35–8.4
shallow sites > 1: 54
shallow sites < 1: 5
7% as threshold, shallow changing at > 2 site(s)
P = 0.046
HR = 6
95% CI: 2.1–17
shallow sites > 2: 46
shallow sites < 2: 13
7% as threshold, shallow changing at > 3 site(s)
P = 0.021
HR = 2.9
95% CI: 0.88–9.6
shallow sites > 3: 14
shallow sites < 3: 45
7% as threshold, shallow changing at > 4 site(s)
P = 0.024
HR = 4.6
95% CI: 0.26–82
shallow sites > 4: 3
shallow sites < 4: 56
8% as threshold, shallow changing at > 0 site(s)
Insufficient group sizes
8% as threshold, shallow changing at > 1 site(s)
P = 0.71
HR = 1.5
95% CI: 0.27–8.3
shallow sites > 1: 55
shallow sites < 1: 4
8% as threshold, shallow changing at > 2 site(s)
P = 0.12
HR = 4.2
95% CI: 1.3–13
shallow sites > 2: 50
shallow sites < 2: 9
8% as threshold, shallow changing at > 3 site(s)
P = 0.058
HR = 2.4
95% CI: 0.77–7.4
shallow sites > 3: 16
shallow sites < 3: 43
8% as threshold, shallow changing at > 4 site(s)
P = 0.024
HR = 4.6
95% CI: 0.26–82
shallow sites > 4: 3
shallow sites < 4: 56
Proportion surviving
Time (days)

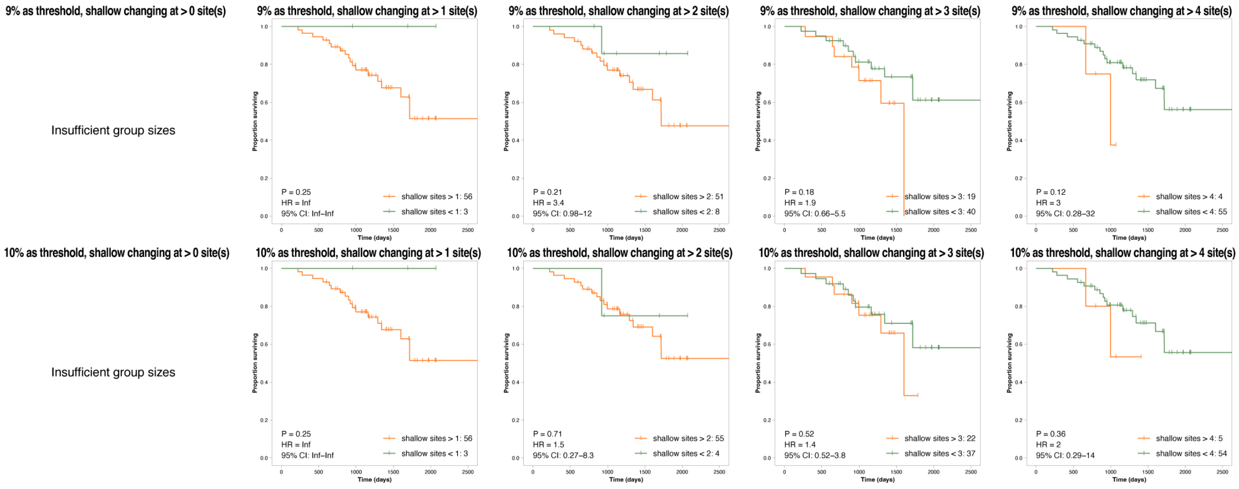

Appendix Figure S6. Kaplan-Meier survival analysis for patients based on the number of sites with shallow DNA methylation changes.

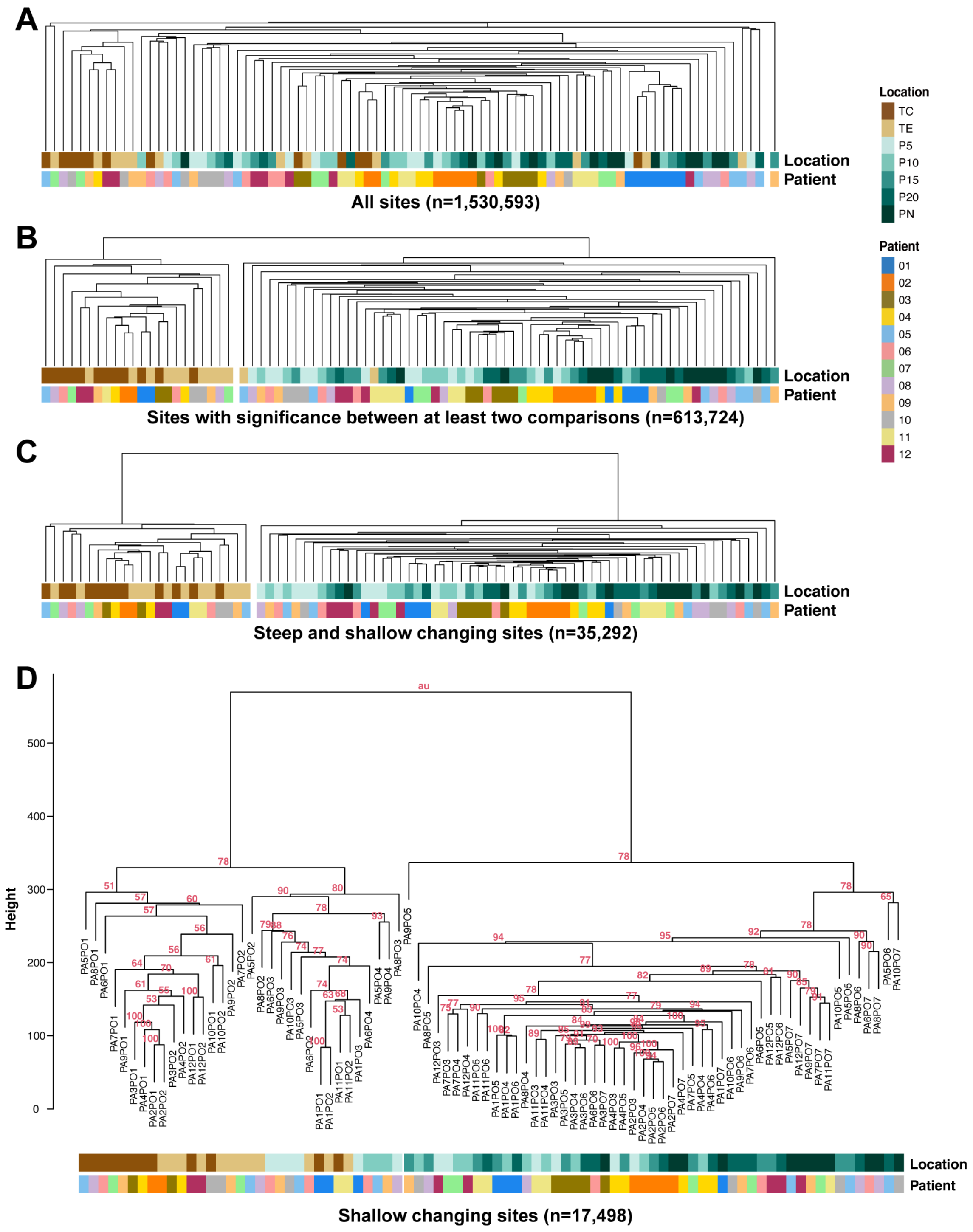
A
Location
Patient
All sites (n=1,530,593)
B
Location
Patient
Sites with significance between at least two comparisons (n=613,724)
C
Location
Patient
Steep and shallow changing sites (n=35,292)
D
Height
au
Location
Patient
Shallow changing sites (n=17,498)
Location
TC
TE
P5
P10
P15
P20
PN
Patient
01
02
03
04
05
06
07
08
09
10
11
12

Appendix Figure S7. Hierarchical clustering based on different sets of CpG sites.

(**A**) Clustering of samples using all ~1.53 million CpG sites did not reveal distinct tumor-normal separation. (**B**) Clustering based on ~610,000 filtered CpGs (with ⩾2 significant differences) showed clearer tumor grouping, though two TE samples clustered with adjacent tissues. (**C**) Clustering using the combined set of steep and shallow changing CpG sites (n = 35,292). (**D**) Cluster stability was assessed using pvclust with multiscale bootstrap resampling; high AU values support the clustering of some adjacent samples (P5/P10) with tumor samples.

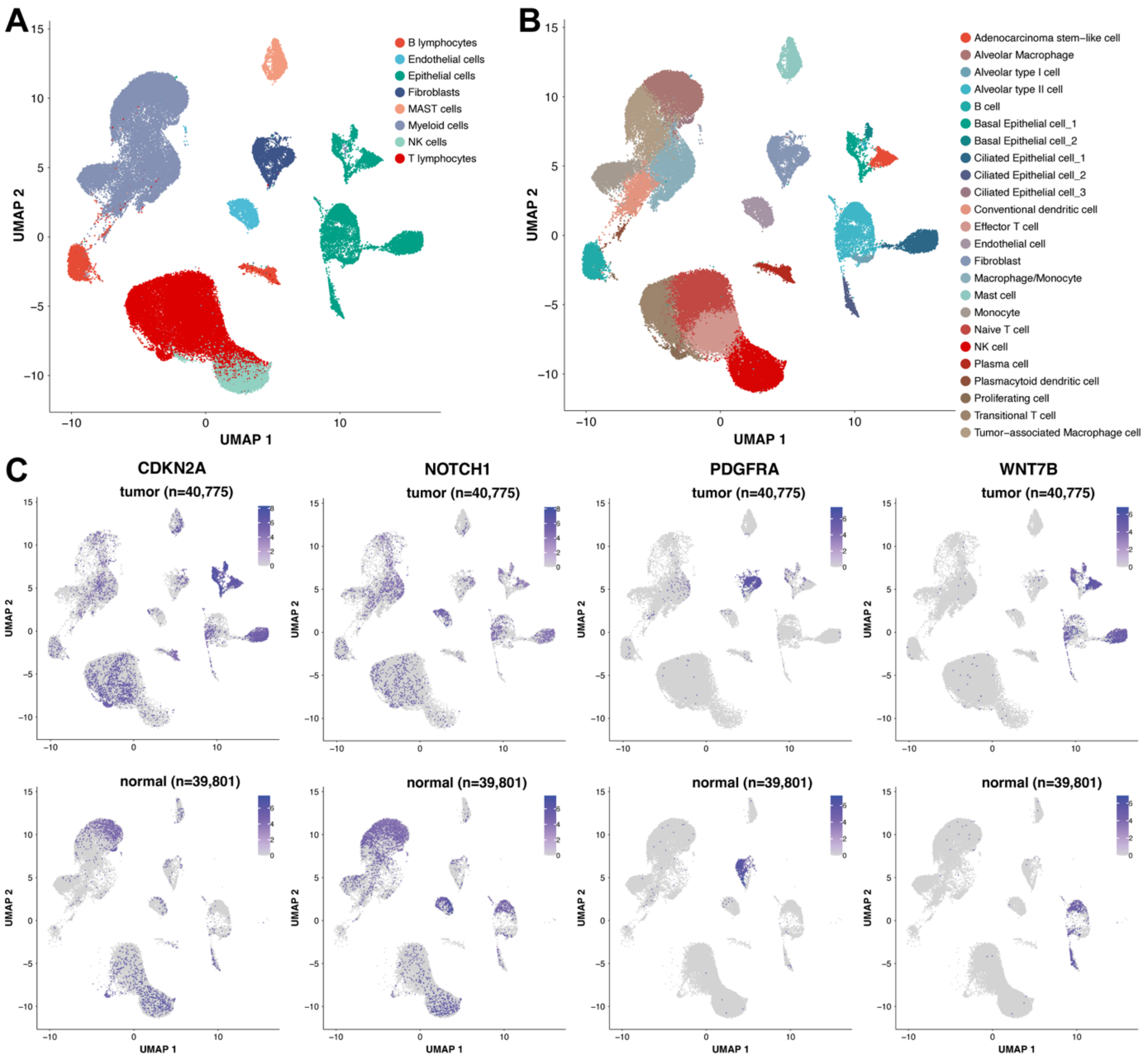

Appendix Figure S8. Single-cell RNA analysis of 80576 cells from 10 LUAD patients.

(**A**) UMAP plot of cells colored by major cell types. (**B**) UMAP plot of cells colored by cell subtypes. (**C**) Expression levels of *CDKN2A*, *NOTCH1*, *PDGFRA*, and *WNT7B* across different cell types in tumor samples (upper four panels) and normal samples (lower four panels). *FZD10* was excluded from the analysis due to low expression.

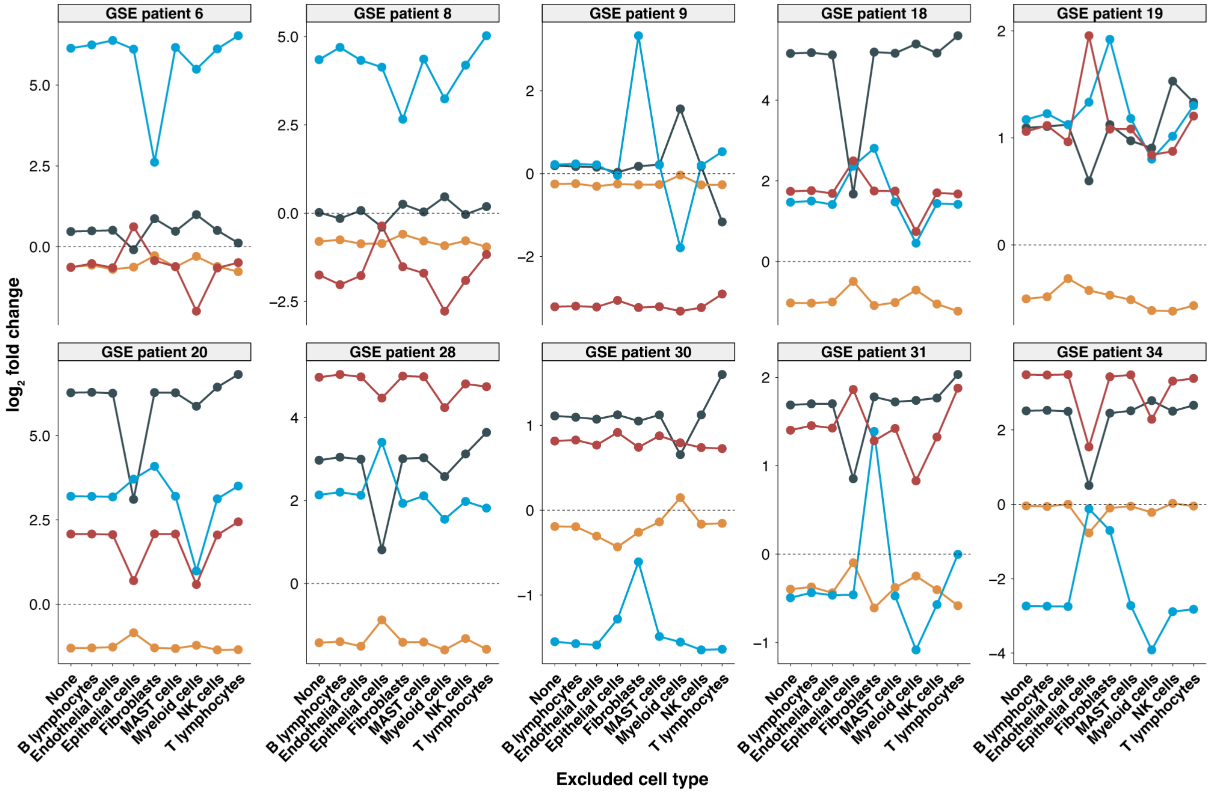

Appendix Figure S9. Impact of cell type exclusion of each patient on target gene log2FC.

Log₂ fold change of aggregated expression differences for target genes between tumor and normal tissues across patients after excluding specific cell types. Pseudo-bulk expression profiles were generated by aggregating single-cell RNA-seq data from different cell types within each patient sample. For each patient, $\log_2$ fold changes between tumor and normal tissues were calculated based on the full dataset ("None") and after systematically removing one cell type at a time: B lymphocytes, endothelial cells, epithelial cells, fibroblasts, myeloid cells, and T lymphocytes. Each line represents the resulting fold-change profile after the exclusion of a specific cell type. Variability across cell-type exclusions reflects the contribution of individual cell populations to observed expression differences of the target genes.

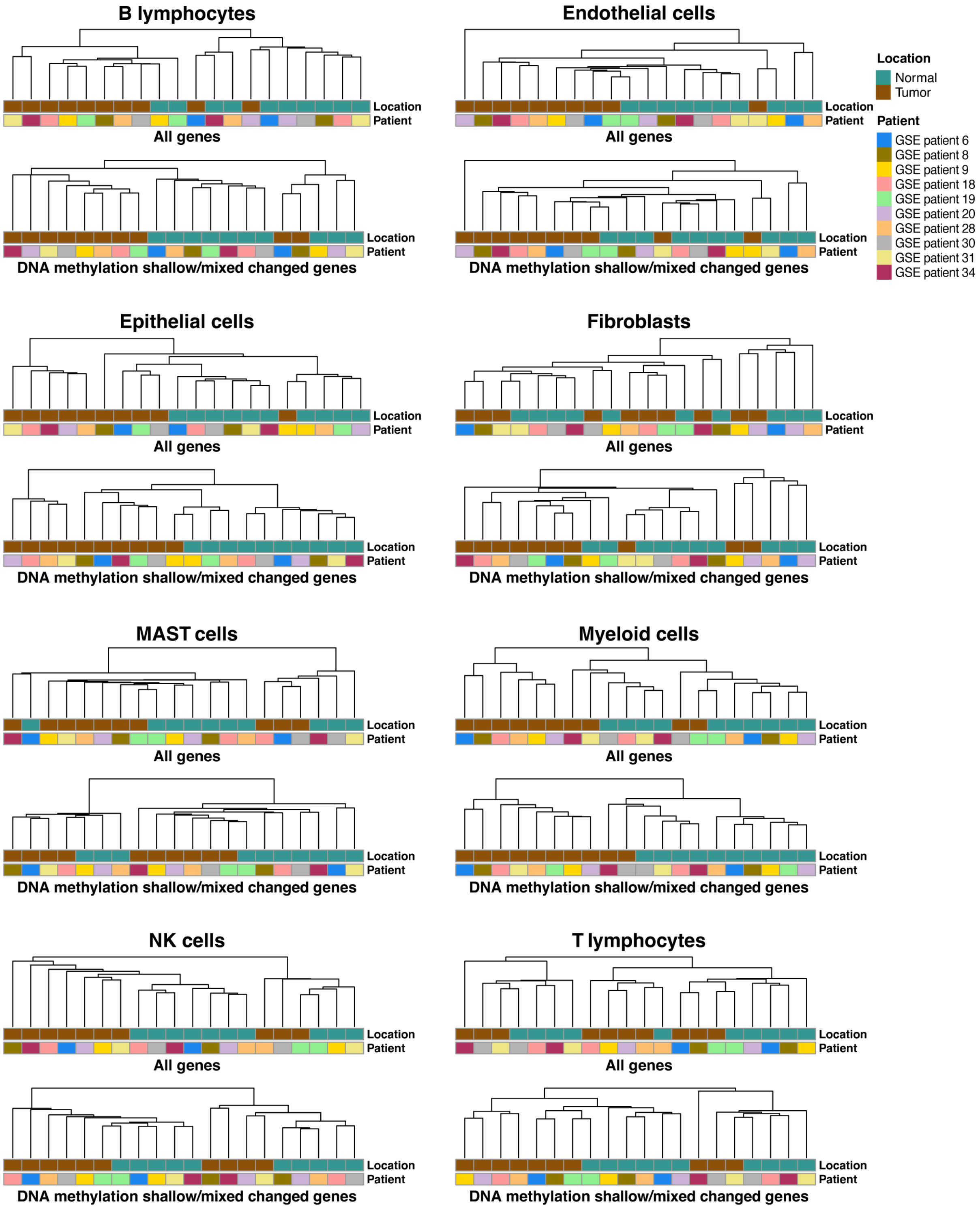
B lymphocytes
Endothelial cells
Epithelial cells
Fibroblasts
MAST cells
Myeloid cells
NK cells
T lymphocytes
Location
Patient
All genes
DNA methylation shallow/mixed changed genes
Location
Normal
Tumor
Patient
GSE patient 6
GSE patient 8
GSE patient 9
GSE patient 18
GSE patient 19
GSE patient 20
GSE patient 28
GSE patient 30
GSE patient 31
GSE patient 34

Appendix Figure S10. Clustering of gene expression profiles in different cell types.

The gene expression patterns of various cell types in tumor and normal samples from different patients are clustered. Each group is a cell type. The upper panel is the situation of all expressed genes, and the lower panel is the expression spectrum clustering of genes with shallow or mixed changing regions.

Appendix Methods

Pipeline of DNA methylation changing trend statistics

Here we explain in more detail the screening process and various standards for the changing trend sites in our study, combined with more examples, in the hope that readers can better understand and reproduce the results, and recognize the rigor of the presented data.

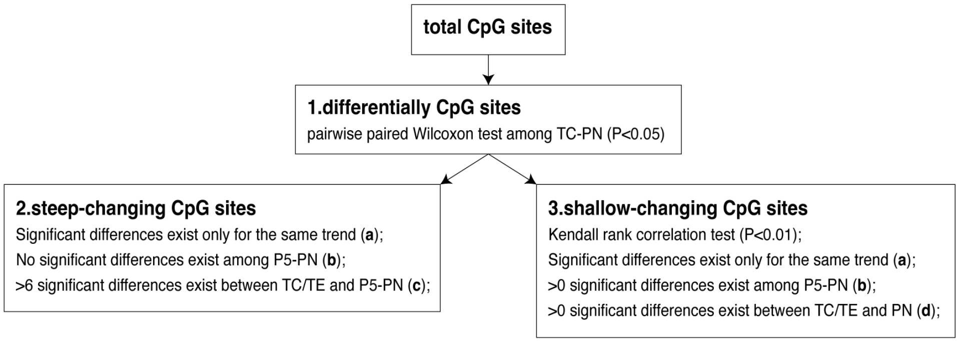

Figure 1

As shown above, our overall screening process can be divided into three steps, including extracting sites with methylation differences from the total CpG sites and performing some feature statistics for these sites in the main text. Then, the steep- and shallow-changing sites were screened among the remaining sites according to different inter-position differences.

Compare between positions and create significant matrices

First, we need to figure out the differences between different sampling positions of each site. We grouped each CpG site by sample location and compared methylation between groups pairwise.

TC<PN	TE<PN	P5<PN	P10<PN	P15<PN	P20<PN	/
TC<P20	TE<P20	P5<P20	P10<P20	P15<P20	/	PN<P20
TC<P15	TE<P15	P5<P15	P10<P15	/	P20<P15	PN<P15
TC<P10	TE<P10	P5<P10	/	P15<P10	P20<P10	PN<P10
TC<P5	TE<P5	/	P10<P5	P15<P5	P20<P5	PN<P5
TC<TE	/	P5<TE	P10<TE	P15<TE	P20<TE	PN<TE
/	TE<TC	P5<TC	P10<TC	P15<TC	P20<TC	PN<TC

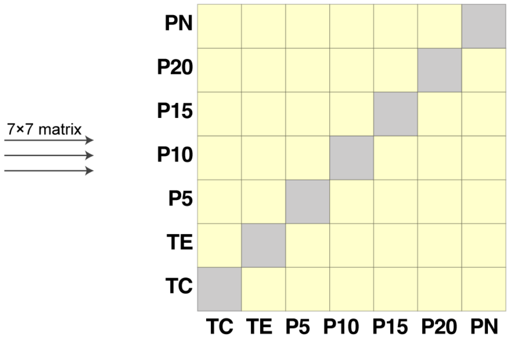

Figure 2

The paired one-tailed Wilcoxon test was used to count the significance with a P<0.05 threshold, and 42 comparations were done for each CpG site (as Fig.2 shows). From the results of the comparison between groups, a 7×7 logical matrix can be formed as the significant matrix, on which each position corresponds to whether the difference between the two groups is significant.

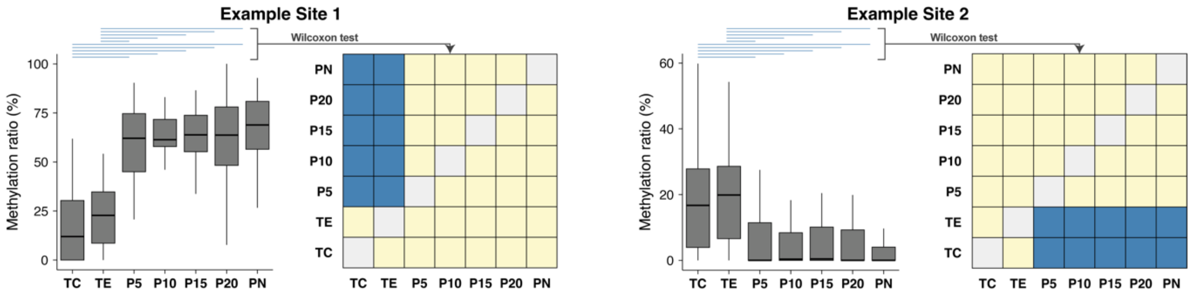

Figure 3

For example, in Fig.3, methylation of Example 1 on TC and TE were significantly less than on P5 to PN, while methylation of Example 2 on TC and TE were significantly higher than on P5 to PN, respectively.

From total CpG sites to differentially-methylated sites

For all CpG sites, many did not have any significant difference in methylation across different locations, and the observation of different characteristics will be affected when these sites are retained. To better extract the location characteristics of the differences, we only retained the CpG sites with at least two significant differences for subsequent analysis, and finally, a total of 613,724 CpG sites were obtained with significance between at least two comparisons.

Examples:

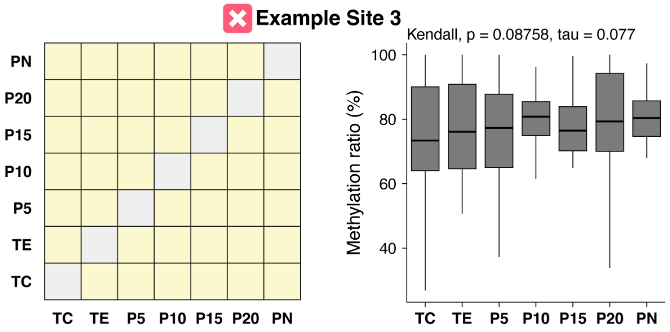

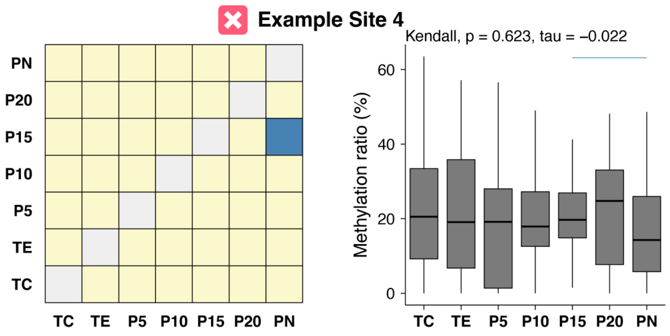

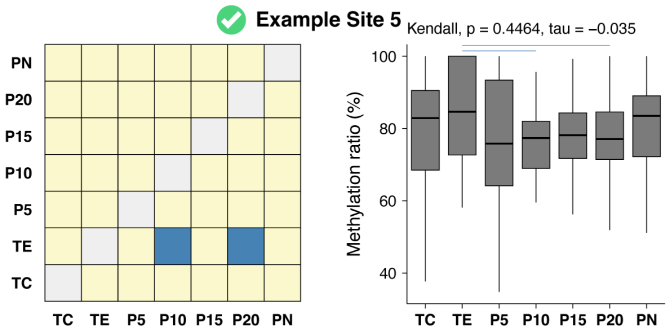

For example, in Fig.4, there was only 0 and 1 significant difference between the positions of Example 3 and Example 4, respectively, while Example 5 had significance on P10<TE and P20<TE. In these three sites, we would keep Example Site 5 for the next part.

From differentially-methylated sites to steep-changing sites

When the screen for steep-changing sites, we first required the differences must occur on the same side, to exclude some sites with fluctuating methylation changes by position order (area A marked in Fig.4A). The steep-changing sites should mainly reflect the difference between tumor tissue and non-tumor tissue, so we required there should be no difference among P5-PN (area B marked in Fig.4B). At the same time, we require sites have no less than 6 significant differences between TC/TE and P5-PN to conform to the trend of steep changes (area C marked in Fig.4C). In the end, we got sites with the steep-changing trend.

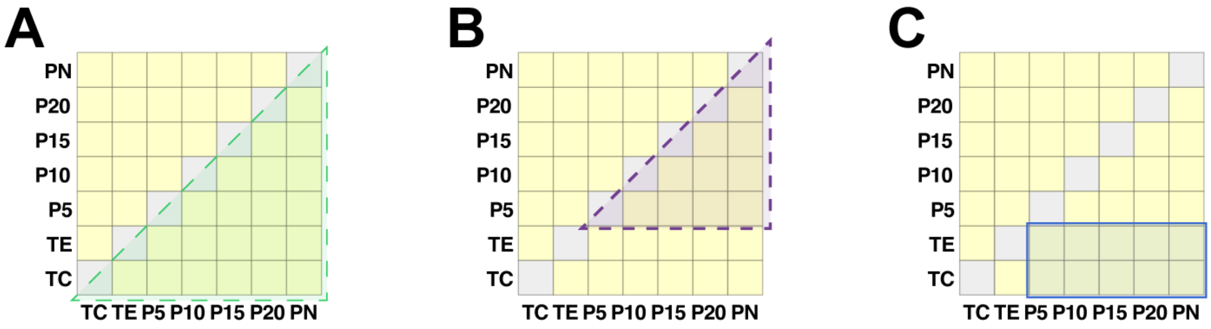

Figure 4

Examples:

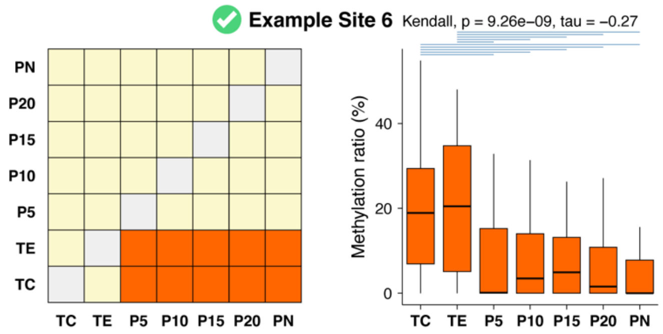

In Example 6, methylations on P5-PN were significantly less than on TC and TE, respectively, counting for 10 significant differences in area C. Besides, all significant differences occurred on the same side and no significance occurred in area B. So, we take this site as a steep-changing CpG site.

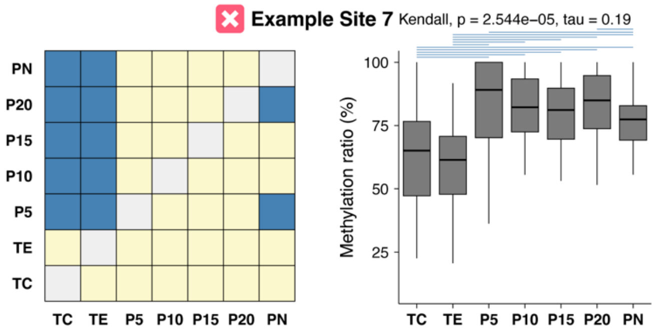

In Example 7, there were significances on both sides, which didn't meet the requirement. Although there are enough differences in area C, indicating methylation on TC and TE were

significantly different from P5 to PN, it is not difficult to see fluctuations in methylation levels between PN and P20 and between PN and P5, inconsistent with the overall trend.

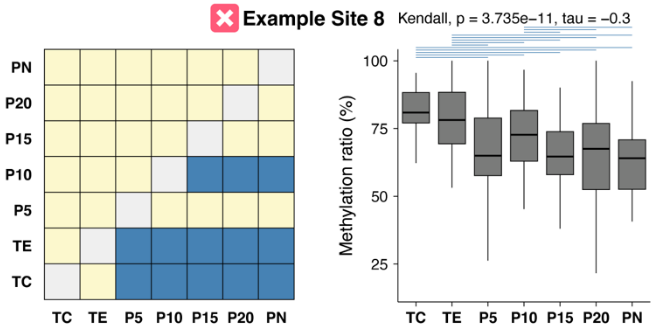

In Example 8, there were significances in area B, which didn’t meet the requirement. Due to the differences between non-tumor samples, it is not difficult to see that the methylation level in the adjacent samples is lower than that of the tumor but higher than that of distant normal tissues. We think this methylation change trend is more similar to shallow-changing, which describes gradual changes.

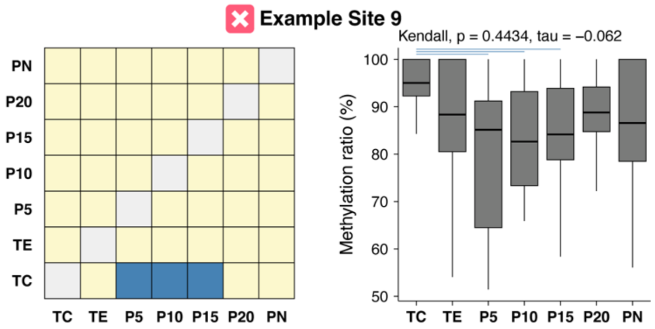

In Example 9, there were not enough significances in area C.

From differentially-methylated sites to shallow-changing sites

When it comes to shallow-changing sites, in the order of TC, TE, P5, P10, P15, P20, and PN, we performed perform ranking-based non-parametric tests, calculated Kendall's rank correlation coefficient of methylation at each significant difference site, and screened with $P<0.01$. Same as steep-changing sites, we screened sites with all significant differences on the same side (area A marked in Fig.5A). For shallow-changing sites, significant changes should be seen between non-

tumor tissues, so we required at least one difference happened among P5-PN (area B marked in Fig.5B). Considering that the shallow-changing sites gradually changed with the distance, we did not require that there were huge differences between TC/TE and P5-PN but only need to ensure that there was difference between TC/TE and the farthest PN (area D marked in Fig.5C). In the end, we got sites with the steep-changing trend.

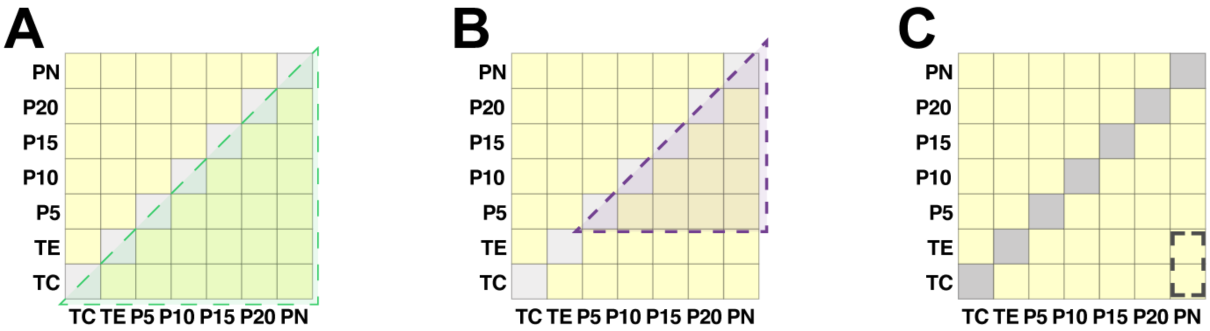

Figure 5

Examples:

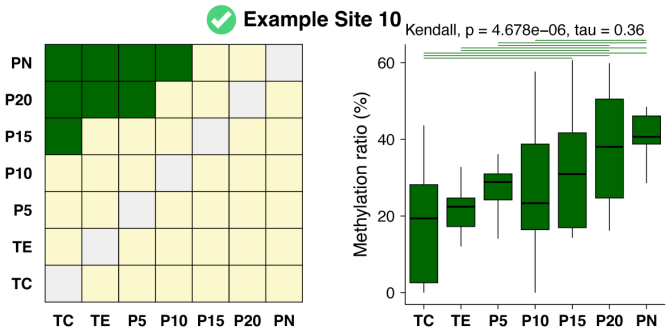

Example 10 is a shallow-changing site. It had a P-value<0.01 in Kendall's correlation test. Methylation on TC was significantly less than on P15 to PN, TE and P5 less than P20 and PN, and P10 less than PN. The difference pattern of the site met the requirement above. So, we take this site as a shallow-changing CpG site.

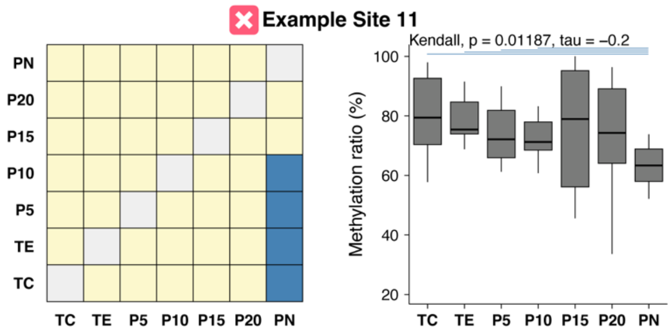

In Example 11, the P-value of the site in Kendall's correlation test is higher than 0.01. Although the rest of the features meet our requirements, it was excluded.

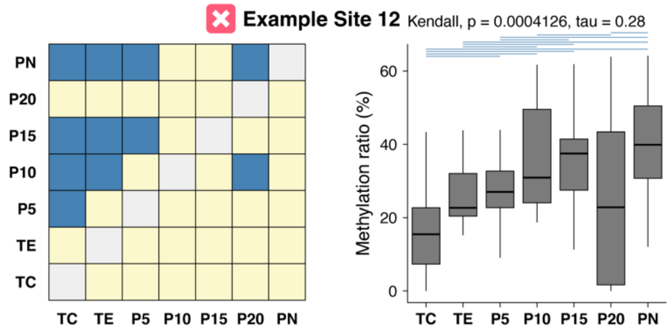

In Example 12, there were differences on both sides, which might reflect potential fluctuations in methylation, and it was excluded.

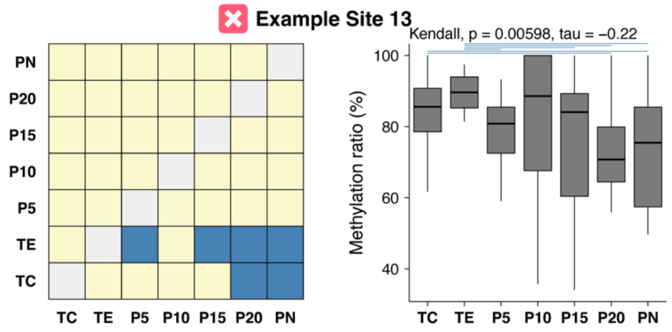

In Example 13, there were no significant changes between non-tumor tissues, so this site was excluded.

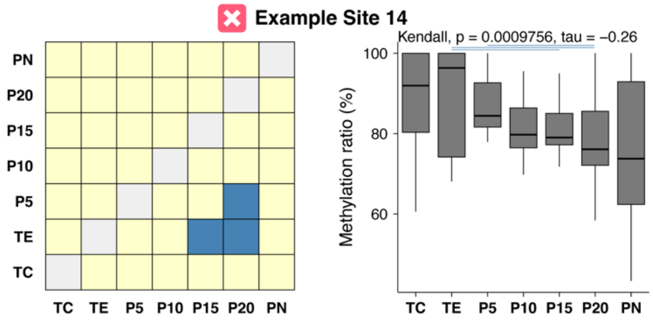

In Example 14, methylation significantly decreased with distance, and there was a significant difference between P5 and P20. However, such points were excluded due to the absence of significant differences between tumor (TC, TE) and distant normal tissue (PN).
