# Supplementary material for: Early-stage lung adenocarcinoma affects DNA methylation and gene expression in adjacent tissues
Source: EMBO Rep. 2025 Nov 3;26(23):5931–58. doi: 10.1038/s44319-025-00612-4 (PMC12678790; doi:10.1038/s44319-025-00612-4)
Supplement: Supplementary file 2 — Table EV1 [file 44319_2025_612_MOESM2_ESM.docx]

**Table EV1.** Clinical and histopathological characteristics of participants in both discovery and validation stages. RUL: right upper lobe; RML: right middle lobe; RLL: right lower lobe; LUL: left upper lobe; LLL: left lower lobe.

| **Discovery stage** | | | | | | | | | |
| --- | --- | --- | --- | --- | --- | --- | --- | --- | --- |
| **Tissues from 12 early LUAD patients** | | | | | | | | | |
| **Sample No.** | **Sex** | **Age of onset** | **Pathology** | **Diameter** | **Smoking history** | **Stage** | **Location** | **Radiology** |  |
| 01 | male | 70 | adenocarcinoma | 1.33 | 0 | IA1 | RUL | GGO |  |
| 02 | male | 39 | adenocarcinoma | 0.75 | 0 | IA1 | LUL | GGO |  |
| 03 | female | 49 | adenocarcinoma | 1.00 | 0 | IA1 | LLL | GGO |  |
| 04 | male | 49 | adenocarcinoma | 2.00 | 0 | IA2 | RUL | GGO |  |
| 05 | male | 52 | adenocarcinoma | 2.20 | 1 | IA3 | RLL | GGO |  |
| 06 | female | 55 | adenocarcinoma | 1.10 | 0 | IA2 | RUL | GGO |  |
| 07 | female | 30 | adenocarcinoma | 1.00 | 0 | IA1 | RLL | GGO |  |
| 08 | female | 31 | adenocarcinoma | 1.30 | 0 | IA2 | LUL | GGO |  |
| 09 | female | 54 | adenocarcinoma | 1.00 | 0 | IA1 | RUL | GGO |  |
| 10 | female | 44 | adenocarcinoma | 0.80 | 0 | IA1 | RUL | GGO |  |
| 11 | female | 53 | adenocarcinoma | 1.74 | 0 | IA2 | LUL | GGO |  |
| 12 | female | 60 | adenocarcinoma | 2.00 | 0 | IA2 | LUL | GGO |  |

| **mRNA validation stage** | | | | | | | | | |
| --- | --- | --- | --- | --- | --- | --- | --- | --- | --- |
| **Tissues from 24 early LUAD patients** | | | | | | | | | |
| **Sample No.** | **Sex** | **Age of onset** | **Pathology** | **Diameter** | **Smoking history** | **Stage** | **Location** | **Radiology** |  |
| 01 | female | 62 | adenocarcinoma | 0.77 | 0 | AIS | LUL | GGO |  |
| 02 | male | 72 | adenocarcinoma | 1.6 | 0 | IA2 | RUL | GGO |  |
| 03 | female | 70 | adenocarcinoma | 0.9 | 0 | IA1 | RUL | GGO |  |
| 04 | female | 64 | adenocarcinoma | 0.7 | 0 | IA1 | RML | GGO |  |
| 05 | female | 57 | adenocarcinoma | 1.2 | 0 | IA2 | RUL | GGO |  |
| 06 | male | 63 | adenocarcinoma | 1.2 | 1 | IA2 | RML | GGO |  |
| 07 | female | 68 | adenocarcinoma | 1.1 | 0 | IA2 | LUL | GGO |  |
| 08 | female | 55 | adenocarcinoma | 1.1 | 0 | IA2 | LLL | GGO |  |
| 09 | male | 85 | adenocarcinoma | 1.2 | 0 | IA2 | LUL | GGO |  |
| 10 | female | 53 | adenocarcinoma | 1.4 | 0 | IA2 | RUL | GGO |  |
| 11 | female | 36 | adenocarcinoma | 1 | 0 | IA1 | RUL | GGO |  |
| 12 | male | 40 | adenocarcinoma | 1.1 | 0 | IA2 | RLL | GGO |  |
| 13 | female | 54 | adenocarcinoma | 1.3 | 0 | IA2 | RUL | GGO |  |
| 14 | female | 54 | adenocarcinoma | 1 | 0 | IA1 | LLL | GGO |  |
| 15 | female | 40 | adenocarcinoma | 0.5 | 0 | IA1 | LLL | GGO |  |
| 16 | female | 46 | adenocarcinoma | 1.8 | 0 | IA2 | RUL | GGO |  |
| 17 | male | 53 | adenocarcinoma | 2.3 | 0 | IA3 | RUL | GGO |  |
| 18 | female | 57 | adenocarcinoma | 1.2 | 0 | IA2 | LUL | GGO |  |
| 19 | female | 44 | adenocarcinoma | 0.9 | 0 | IA1 | RUL | GGO |  |
| 20 | male | 66 | adenocarcinoma | 2.2 | 0 | IA3 | LUL | GGO |  |
| 21 | female | 63 | adenocarcinoma | 1.9 | 1 | IA2 | LUL | GGO |  |
| 22 | female | 68 | adenocarcinoma | 2.8 | 0 | IA3 | LLL | GGO |  |
| 23 | female | 44 | adenocarcinoma | 0.8 | 0 | IA1 | RML | GGO |  |
| 24 | male | 67 | adenocarcinoma | 2.4 | 1 | IA3 | LUL | GGO |  |

| **Prognosis validation stage** | | | | | | | | | |
| --- | --- | --- | --- | --- | --- | --- | --- | --- | --- |
| **FFPE from 59 LUAD patients** | | | | | | | | | |
| **Sample No.** | **Sex** | **Age of onset** | **Pathology** | **Diameter** | **Smoking history** | **Stage** | **Location** | **Radiology** |  |
| 01 | male | 55 | adenocarcinoma | 2.3 | NA | IA3 | NA | NA |  |
| 02 | male | 45 | adenocarcinoma | 0.8 | NA | IA | NA | NA |  |
| 03 | female | 63 | adenocarcinoma | 1.1 | NA | IA | RUL | NA |  |
| 04 | male | 53 | adenocarcinoma | 1.4 | NA | IA | RUL | NA |  |
| 05 | female | 59 | adenocarcinoma | 2 | NA | IA | NA | NA |  |
| 06 | female | 39 | adenocarcinoma | 3 | NA | IA3 | RUL | NA |  |
| 07 | male | 60 | adenocarcinoma | 2.1 | NA | IA2 | RML | NA |  |
| 08 | female | 68 | adenocarcinoma | 2.8 | NA | IA3 | RUL | NA |  |
| 09 | male | 50 | adenocarcinoma | 2.8 | NA | NA | RUL | NA |  |
| 10 | female | 53 | adenocarcinoma | 2.8 | NA | IVA | LUL | NA |  |
| 11 | female | 47 | adenocarcinoma | 1.8 | NA | IIIA | LLL | NA |  |
| 12 | female | 60 | adenocarcinoma | 4.5 | NA | IIA | RUL | NA |  |
| 13 | female | 50 | adenocarcinoma | 1.2 | NA | IA2 | LLL | NA |  |
| 14 | female | 82 | adenocarcinoma | 2.2 | NA | IA3 | LLL | NA |  |
| 15 | male | 65 | adenocarcinoma | 4.7 | NA | IIIA | LUL | NA |  |
| 16 | female | 73 | adenocarcinoma | 3.3 | NA | IIIA | RML | NA |  |
| 17 | female | 52 | adenocarcinoma | 1.9 | NA | IA2 | RUL | NA |  |
| 18 | female | 55 | adenocarcinoma | 1 | NA | IA1 | LUL | NA |  |
| 19 | female | 59 | adenocarcinoma | 3 | NA | IA3 | RUL | NA |  |
| 20 | female | 66 | adenocarcinoma | 2.8 | NA | IB | RUL | NA |  |
| 21 | male | 70 | adenocarcinoma | 1.3 | NA | IA2 | LLL | NA |  |
| 22 | female | 43 | adenocarcinoma | 0.6 | NA | IA1 | RML | NA |  |
| 23 | male | 69 | adenocarcinoma | 1 | NA | IA1 | RUL | NA |  |
| 24 | female | 60 | adenocarcinoma | 0.8 | NA | IA1 | RUL | NA |  |
| 25 | male | 73 | adenocarcinoma | NA | NA | IA | RML | NA |  |
| 26 | female | 30 | adenocarcinoma | 1.2 | NA | IA2 | LUL | NA |  |
| 27 | male | 77 | adenocarcinoma | 4.1 | NA | IIA | LUL | NA |  |
| 28 | female | 41 | adenocarcinoma | 1 | NA | IA1 | RLL | NA |  |
| 29 | male | 72 | adenocarcinoma | 4.4 | NA | IIB | LLL | NA |  |
| 30 | male | 67 | adenocarcinoma | 7.5 | NA | IIIA | LLL | NA |  |
| 31 | female | 55 | adenocarcinoma | 1.8 | NA | IA2 | RML | NA |  |
| 32 | female | 61 | adenocarcinoma | 1.1 | NA | IA2 | RLL | NA |  |
| 33 | male | 58 | adenocarcinoma | 1.8 | NA | IA2 | LUL | NA |  |
| 34 | female | 67 | adenocarcinoma | 0.6 | NA | IA1 | RUL | NA |  |
| 35 | male | 65 | adenocarcinoma | 0.5 | NA | IB | LUL | NA |  |
| 36 | male | 78 | adenocarcinoma | 0.7 | NA | IIB | LUL | NA |  |
| 37 | female | 33 | adenocarcinoma | 0.8 | NA | IA1 | LUL | NA |  |
| 38 | female | 71 | adenocarcinoma | 1 | NA | IA1 | RUL | NA |  |
| 39 | male | 68 | adenocarcinoma | 2.8 | NA | IIB | LUL | NA |  |
| 40 | female | 59 | adenocarcinoma | 1 | NA | IA1 | RLL | NA |  |
| 41 | female | 77 | adenocarcinoma | 2.3 | NA | IA3 | RUL | NA |  |
| 42 | male | 70 | adenocarcinoma | 2 | NA | IIIA | RLL | NA |  |
| 43 | male | 74 | adenocarcinoma | NA | NA | IV | LUL | NA |  |
| 44 | male | 63 | adenocarcinoma | 1.3 | NA | IA2 | LUL | NA |  |
| 45 | female | 44 | adenocarcinoma | 1.8 | NA | IA2 | RUL | NA |  |
| 46 | female | 64 | adenocarcinoma | 3.4 | NA | IIB | LUL | NA |  |
| 47 | female | 62 | adenocarcinoma | 1.1 | NA | IA2 | RUL | NA |  |
| 48 | female | 61 | adenocarcinoma | 1.8 | NA | IA2 | RUL | NA |  |
| 49 | male | 51 | adenocarcinoma | 3.1 | NA | IIIB | LLL | NA |  |
| 50 | male | 66 | adenocarcinoma | 4.3 | NA | IIIA | LLL | NA |  |
| 51 | male | 54 | adenocarcinoma | 3.7 | NA | IIB | LLL | NA |  |
| 52 | female | 69 | adenocarcinoma | 2.2 | NA | IA3 | LLL | NA |  |
| 53 | male | 73 | adenocarcinoma | 1.2 | NA | IA2 | LUL | NA |  |
| 54 | male | 72 | adenocarcinoma | 0.83 | NA | IA1 | RLL | NA |  |
| 55 | female | 68 | adenocarcinoma | 2.1 | NA | IA3 | RML | NA |  |
| 56 | female | 48 | adenocarcinoma | 0.7 | NA | IA1 | RUL | NA |  |
| 57 | female | 77 | adenocarcinoma | 1.9 | NA | IA2 | RUL | NA |  |
| 58 | female | 50 | adenocarcinoma | 1.1 | NA | IA2 | LUL | NA |  |
| 59 | male | 57 | adenocarcinoma | 1.5 | NA | IA2 | LUL | NA |  |
