# Supplementary material for: Early-stage lung adenocarcinoma affects DNA methylation and gene expression in adjacent tissues
Source: EMBO Rep. 2025 Nov 3;26(23):5931–58. doi: 10.1038/s44319-025-00612-4 (PMC12678790; doi:10.1038/s44319-025-00612-4)
Supplement: Supplementary file 3 — Table EV2 [file 44319_2025_612_MOESM3_ESM.docx]

**Table EV2.** Primers used for RT-qPCR and BSAS targeting specific genes.

| **mRNA validation stage (RT-qPCR)** | | | |
| --- | --- | --- | --- |
| **Gene name** | **Forward primer** | **Reverse primer** | **Target length** |
| ACTB | CTTCCAGCCTTCCTTCCTGG | CTGTGTTGGCGTACAGGTCT | 110 |
| CDKN2A | ATGGAGCCTTCGGCTGACT | GTAACTATTCGGTGCGTTGGG | 108 |
| FZD10 | GAGAAGCTCATGGTGCGTATC | TAAAAGTAGCAGGCGATCACA | 80 |
| NOTCH1 | GATGCCAGGACCCCAACC | CACAGCTGCAGGCATAGTCT | 102 |
| PDGFRA | CGACAGCAGACAGGGCTTTA | CTTTCCTTTGACGGTGGCCT | 73 |
| WNT7B | GAAGCAGGGCTACTACAACCA | CGGCCTCATTGTTATGCAGGT | 155 |

| **Prognosis validation stage (BSAS)** | | | |
| --- | --- | --- | --- |
| **Gene name** | **Forward primer** | **Reverse primer** | **Target length** |
| FZD10 | GGGATTTATTATAAAAGGAAGAGAAGAT | TACCTAACCTCCAACTTTACCTCTC | 195 |
| NOTCH1 | TTGTAGGGGTTGGGGGTATA | CCCTAAATCAAAATCCTAACACAAC | 223 |
| WNT7B | TTGGTTTATTTTTTTAAGTTTTT | AAACCAAACCAAAACTATATAACTCC | 203 |
