# Supplementary material for: Early-stage lung adenocarcinoma affects DNA methylation and gene expression in adjacent tissues
Source: EMBO Rep. 2025 Nov 3;26(23):5931–58. doi: 10.1038/s44319-025-00612-4 (PMC12678790; doi:10.1038/s44319-025-00612-4)
Supplement: Supplementary file 4 — Table EV3 [file 44319_2025_612_MOESM4_ESM.docx]

**Table EV3.** Methodological comparison with the previous study.

|  | Jia et. al, 2021 | Wu et. al, 2024 |
| --- | --- | --- |
| Samples | 105 samples  (7 positions of 15 patients) | 84 samples  (7 positions of 12 patients) |
| Total sites | >500,000 CpG sites (10×) | >1,500,000 CpG sites (15×) |
| Methods | 1. find DMC: β_TC_ v.s. β_PN_, $FDR<0.05$and mean $\Delta(\beta_{\mathrm{TC}}-\beta_{\mathrm{TE}})>0.2$;  2. judge change trend:  $\frac{\beta_{\mathrm{TC}}-\beta_{\mathrm{TE}}}{\beta_{\mathrm{TE}}-\beta_{P5}}\geq1$: shallow site;  $\frac{\beta_{\mathrm{TC}}-\beta_{\mathrm{TE}}}{\beta_{\mathrm{TE}}-\beta_{P5}}<1$: steep site. | detailed in Supplementary Information |
| Featured sites | 1,657 DMC and 713 DMR | 613,724 CpG sites (significance>1) |
| Steep-changing | 1,469 CpG sites and 636 regions | 17,794 CpG sites and 755 regions |
| Shallow-changing | 188 CpG sites and 77 regions | 17,498 CpG sites and 362 regions |
