# Supplementary material for: Early-stage lung adenocarcinoma affects DNA methylation and gene expression in adjacent tissues
Source: EMBO Rep. 2025 Nov 3;26(23):5931–58. doi: 10.1038/s44319-025-00612-4 (PMC12678790; doi:10.1038/s44319-025-00612-4)
Supplement: Supplementary file 9 — Expanded View Figures [file 44319_2025_612_MOESM9_ESM.pdf]

Expanded View Figures

Figure EV1. Schematic diagrams illustrating the statistical methods employed.

(**A**) Illustrations of spatial autocorrelation, featuring three typical distribution examples. (**B**) Illustration of the Kendall rank correlation coefficient. All points within the gray area are concordant, while those in the white area are discordant to the reference point. This example contains 395 concordant point pairs and 40 discordant pairs, yielding a Kendall rank correlation coefficient of 0.816. (**C**) The significantly different CpG site at chr1:1,138,200 is associated with no other significant sites within a 30 nt range. (**D**) Boxplots illustrate the methylation levels of the site chr1:1,138,200 across different locations, while matrices display the significantly associated locations for each CpG site. (**E**) The significantly different CpG site at chr2:42,905,798 is associated with three other significant sites within a 30 nt range. (**F**) Boxplots illustrate the methylation levels of these four sites across different locations, while matrices display the significantly associated locations for each CpG site. The methylation patterns of these sites are analogous, with chr2:42,905,798, chr2:42,905,801, and chr2:42,905,812 exhibiting comparable Moran's *I* values. The matrices of these three surrounding sites are respectively compared with the calculated cosine similarity of chr2:42,905,798, and the results are shown on the blue lines. Boxplots present with the median as the center, box bounds as the 25th and 75th percentiles, and whiskers extending to the minimum and maximum values within 1.5×IQR ($n = 12$). Outliers beyond 1.5×IQR are plotted as individual points.

© The Author(s)

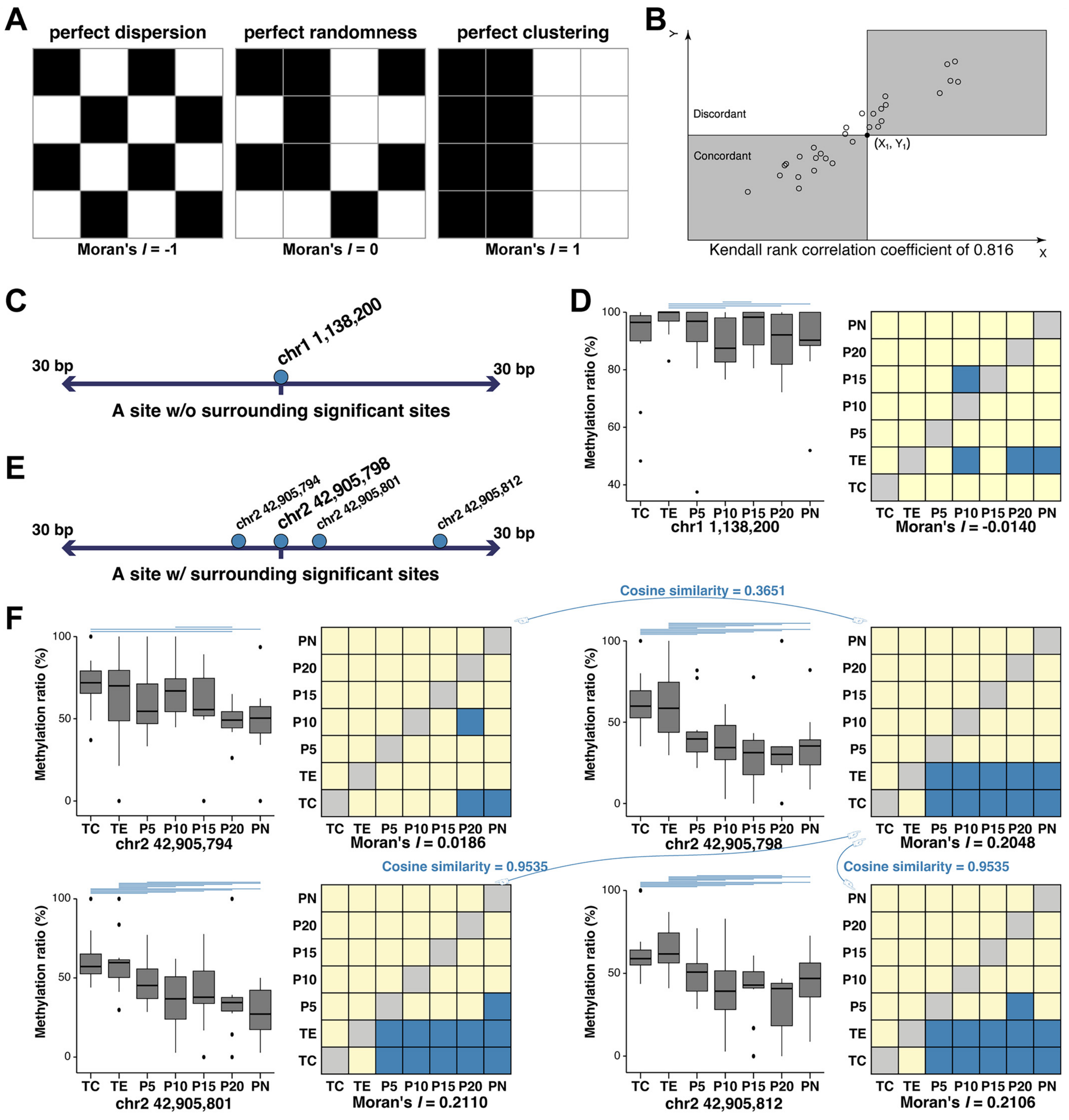
A
perfect dispersion
perfect randomness
perfect clustering
Moran's I = -1
Moran's I = 0
Moran's I = 1
B
Discordant
Concordant
(X1, Y1)
Kendall rank correlation coefficient of 0.816
C
30 bp
chr1 1,138,200
30 bp
A site w/o surrounding significant sites
D
Methylation ratio (%)
chr1 1,138,200
Moran's I = -0.0140
E
30 bp
chr2 42,905,794
chr2 42,905,798
chr2 42,905,801
chr2 42,905,812
30 bp
A site w/ surrounding significant sites
F
Cosine similarity = 0.3651
chr2 42,905,794
Moran's I = 0.0186
chr2 42,905,798
Moran's I = 0.2048
Cosine similarity = 0.9535
Cosine similarity = 0.9535
chr2 42,905,801
Moran's I = 0.2110
chr2 42,905,812
Moran's I = 0.2106
TC TE P5 P10 P15 P20 PN

 © The Author(s)

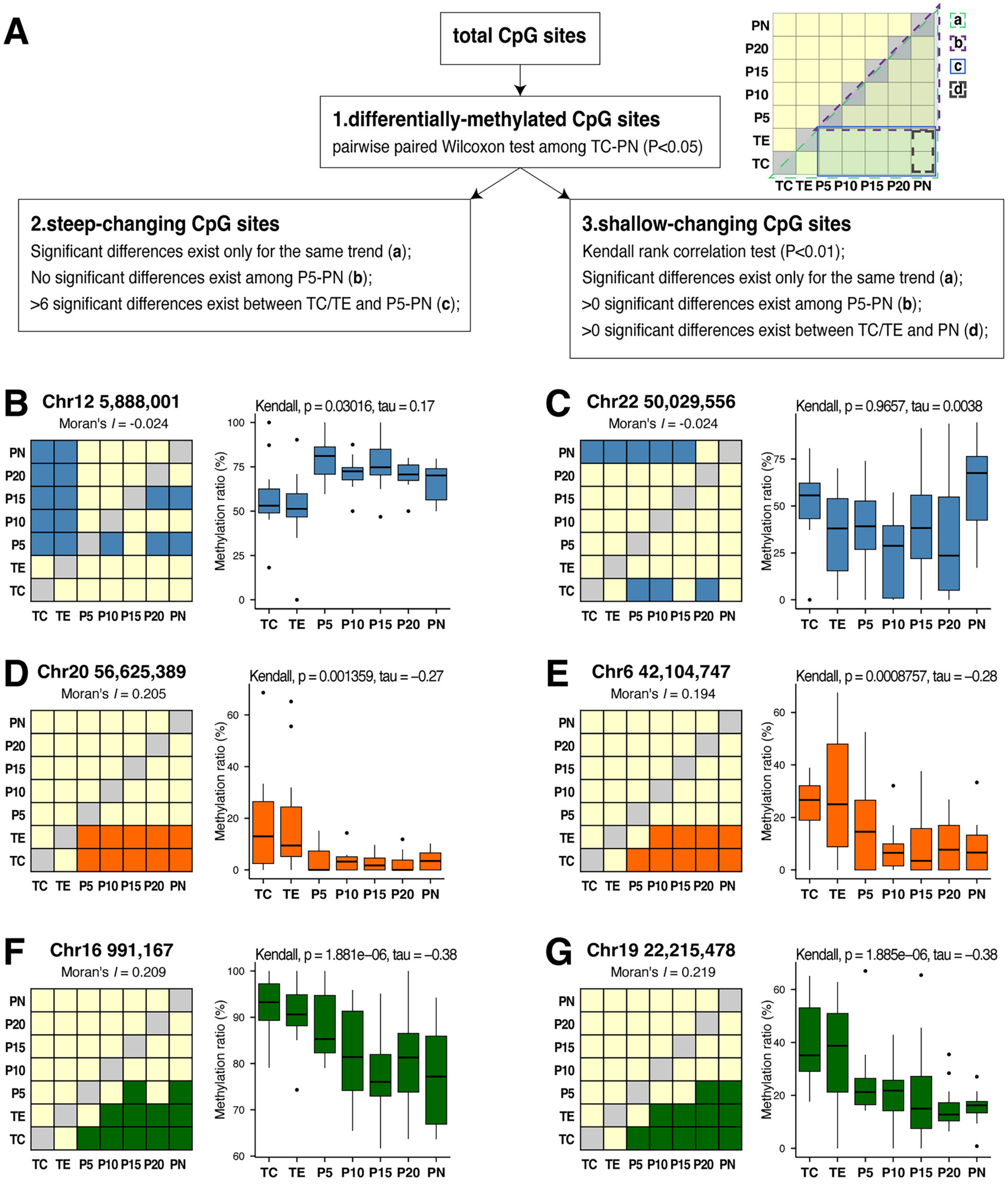

Figure EV2. Classification process for methylation change trends and site examples.

(**A**) The screening process and specific criteria for identifying steep- and shallow-changing sites are outlined. Additional details and examples of criteria for judgment can be found in Appendix Methods. (**B**, **C**) Two differentially methylated CpG sites were excluded for failing to meet the specified criteria. (**D**, **E**) Examples of two steep-changing CpG sites are presented. (**F**, **G**) Examples of two shallow-changing CpG sites are presented. Boxplots present with the median as the center, box bounds as the 25th and 75th percentiles, and whiskers extending to the minimum and maximum values within 1.5×IQR ($n = 12$). Outliers beyond 1.5×IQR are plotted as individual points.

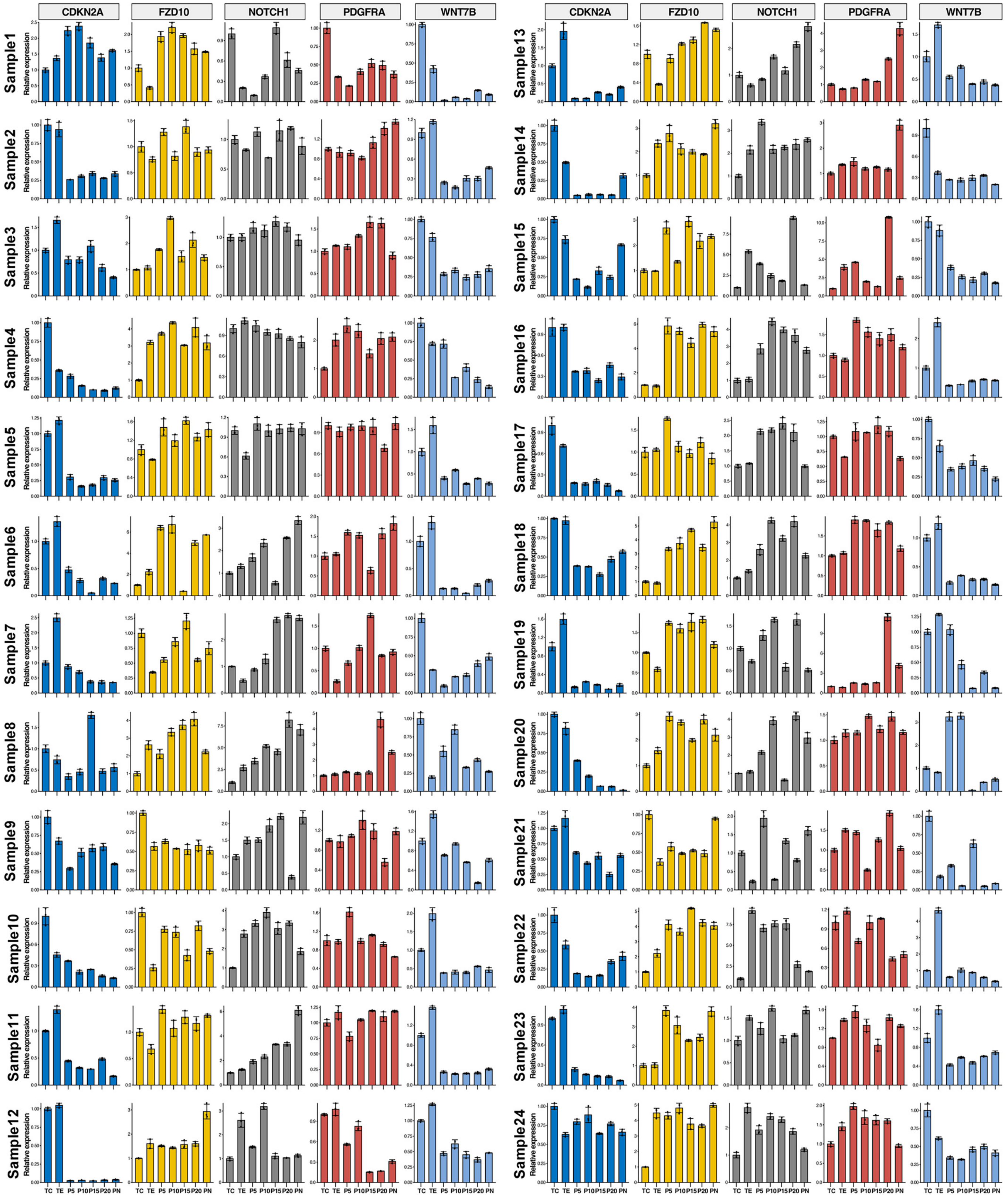

Figure EV3. Relative gene expression at different locations in each sample measured by RT-qPCR.

mRNA expression of five genes from 24 patients are presented. Relative expression levels were normalized to the reference gene *ACTB* and standardized to TC's relative expression for comparison, with error bars indicating the mean ± standard deviation (SD) from three replicates.

 © The Author(s)

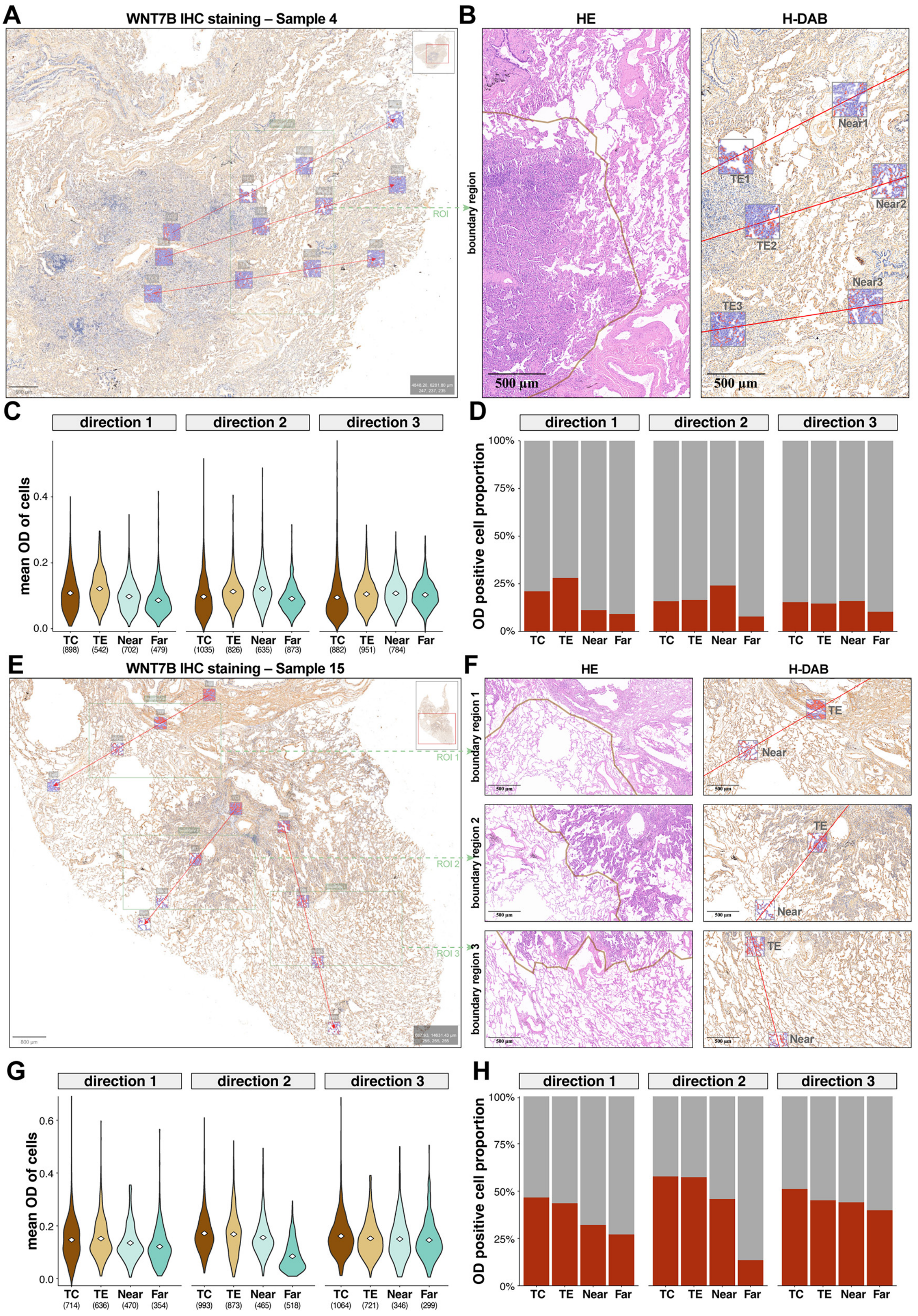
A
WNT7B IHC staining – Sample 4
ROI
B
HE
H-DAB
boundary region
500 µm
Near1
TE1
Near2
TE2
Near3
TE3
C
direction 1
direction 2
direction 3
mean OD of cells
0.0
0.2
0.4
TC (898) TE (542) Near (702) Far (479)
TC (1035) TE (826) Near (635) Far (873)
TC (882) TE (951) Near (784) Far
D
direction 1
direction 2
direction 3
OD positive cell proportion
0%
25%
50%
75%
100%
TC TE Near Far
E
WNT7B IHC staining – Sample 15
ROI 1
ROI 2
ROI 3
F
HE
H-DAB
boundary region 1
boundary region 2
boundary region 3
TE
Near
G
direction 1
direction 2
direction 3
mean OD of cells
0.0
0.2
0.4
0.6
TC (714) TE (636) Near (470) Far (354)
TC (993) TE (873) Near (465) Far (518)
TC (1064) TE (721) Near (346) Far (299)
H
direction 1
direction 2
direction 3
OD positive cell proportion
0%
25%
50%
75%
100%
TC TE Near Far

© The Author(s)

Figure EV4. WNT7B protein expression profiles of Patient 4 and Patient 15.

(**A**) Representative IHC staining of WNT7B protein in LUAD Sample 4. FFPE tumor tissue section was stained using WNT7B antibody. Red lines marked three directions from the tumor core to adjacent tissues, and 300μm-squared regions of interest (ROIs) of the tumor core (TC), tumor edge (TE), and adjacent tissue at different distances (P5–Near and P5–Far) on each direction were selected as for optical density (OD) analysis. (**B**) A larger ROI covering three pairs of TE and P5-Near regions was selected for the local magnification display of the boundary region in Sample 4. HE and H-DAB staining images showed the visual boundary of the tumor and corresponding DAB signals in TE and P5-Near regions. (**C**) Violin plots showing the distribution of mean OD per cell for each region (TC, TE, Near, and Far) in three directions of Sample 4 and cell numbers of ROIs were shown below. (**D**) Proportion of OD-positive cells in each region and direction, based on predefined OD thresholds described in the Methods. (**E**) Representative IHC staining of WNT7B protein in LUAD Sample 15. FFPE tumor tissue section was stained using WNT7B antibody. Red lines marked three directions from the tumor core to adjacent tissues, and 300μm-squared regions of interest (ROIs) of the tumor core (TC), tumor edge (TE), and adjacent tissue at different distances (P5–Near and P5–Far) on each direction were selected as for optical density (OD) analysis. (**F**) Three larger ROIs covering the corresponding TE and P5-Near regions were selected for local magnification display of the boundary region in Sample 15. HE and H-DAB staining images showed the visual boundary of the tumor and corresponding DAB signals in TE and P5-Near regions. (**G**) Violin plots showing the distribution of mean OD per cell for each region (TC, TE, Near, and Far) in three directions of Sample 15 and cell numbers of ROIs were shown below. (**H**) Proportion of OD-positive cells in each region and direction in Sample 15.

 © The Author(s)

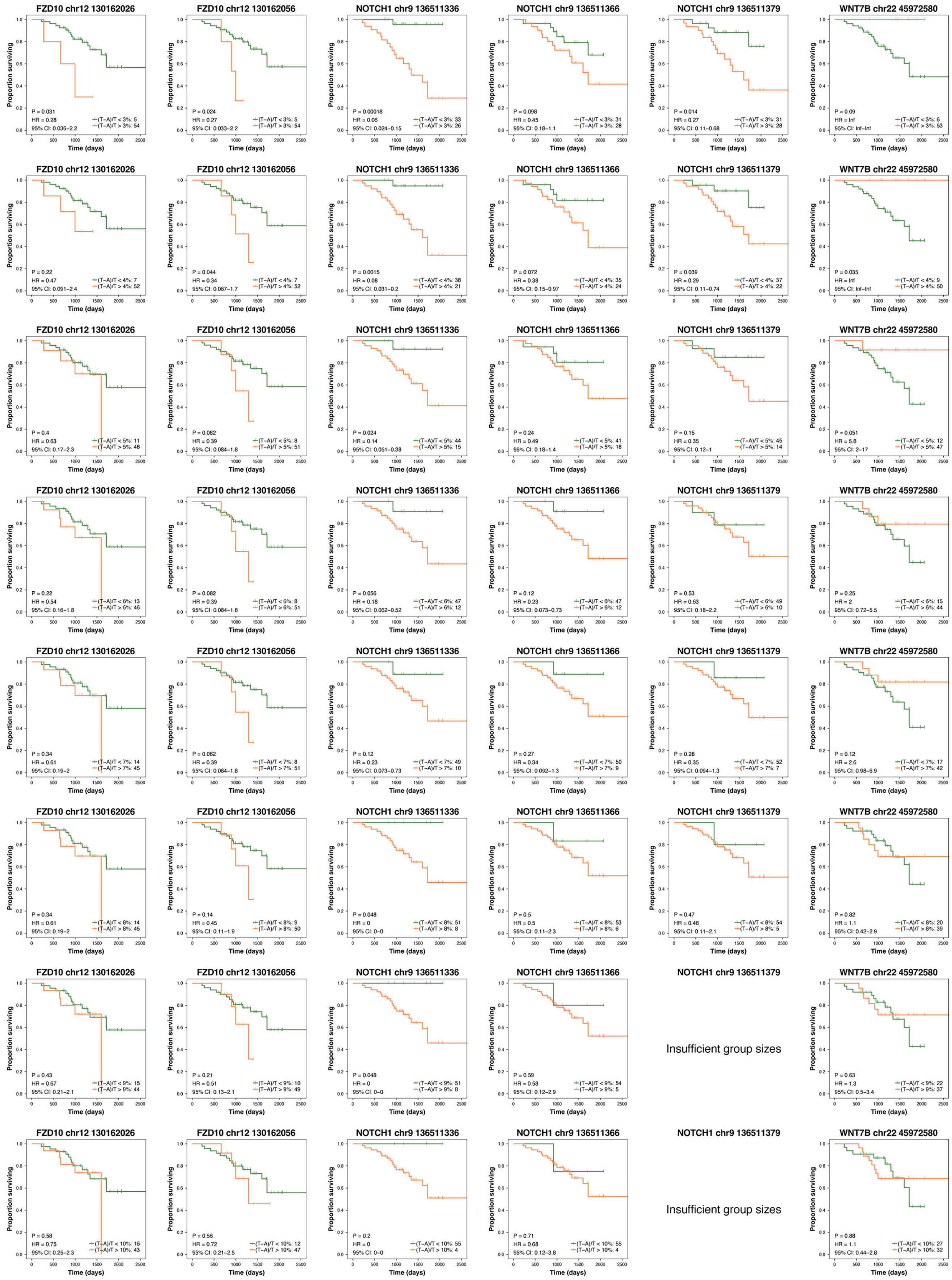
FZD10 chr12 130162026
P = 0.031
HR = 0.28
95% CI: 0.036–2.2
(T−A)/T < 3%: 5
(T−A)/T > 3%: 54
FZD10 chr12 130162056
P = 0.024
HR = 0.27
95% CI: 0.033–2.2
(T−A)/T < 3%: 5
(T−A)/T > 3%: 54
NOTCH1 chr9 136511336
P = 0.00018
HR = 0.06
95% CI: 0.024–0.15
(T−A)/T < 3%: 33
(T−A)/T > 3%: 26
NOTCH1 chr9 136511366
P = 0.098
HR = 0.45
95% CI: 0.18–1.1
(T−A)/T < 3%: 31
(T−A)/T > 3%: 28
NOTCH1 chr9 136511379
P = 0.014
HR = 0.27
95% CI: 0.11–0.68
(T−A)/T < 3%: 31
(T−A)/T > 3%: 28
WNT7B chr22 45972580
P = 0.09
HR = Inf
95% CI: Inf–Inf
(T−A)/T < 3%: 6
(T−A)/T > 3%: 53
FZD10 chr12 130162026
P = 0.22
HR = 0.47
95% CI: 0.091–2.4
(T−A)/T < 4%: 7
(T−A)/T > 4%: 52
FZD10 chr12 130162056
P = 0.044
HR = 0.34
95% CI: 0.067–1.7
(T−A)/T < 4%: 7
(T−A)/T > 4%: 52
NOTCH1 chr9 136511336
P = 0.0015
HR = 0.08
95% CI: 0.031–0.2
(T−A)/T < 4%: 38
(T−A)/T > 4%: 21
NOTCH1 chr9 136511366
P = 0.072
HR = 0.38
95% CI: 0.15–0.97
(T−A)/T < 4%: 35
(T−A)/T > 4%: 24
NOTCH1 chr9 136511379
P = 0.039
HR = 0.29
95% CI: 0.11–0.74
(T−A)/T < 4%: 37
(T−A)/T > 4%: 22
WNT7B chr22 45972580
P = 0.035
HR = Inf
95% CI: Inf–Inf
(T−A)/T < 4%: 9
(T−A)/T > 4%: 50
FZD10 chr12 130162026
P = 0.4
HR = 0.63
95% CI: 0.17–2.3
(T−A)/T < 5%: 11
(T−A)/T > 5%: 48
FZD10 chr12 130162056
P = 0.082
HR = 0.39
95% CI: 0.084–1.8
(T−A)/T < 5%: 8
(T−A)/T > 5%: 51
NOTCH1 chr9 136511336
P = 0.024
HR = 0.14
95% CI: 0.051–0.38
(T−A)/T < 5%: 44
(T−A)/T > 5%: 15
NOTCH1 chr9 136511366
P = 0.24
HR = 0.49
95% CI: 0.18–1.4
(T−A)/T < 5%: 41
(T−A)/T > 5%: 18
NOTCH1 chr9 136511379
P = 0.15
HR = 0.35
95% CI: 0.12–1
(T−A)/T < 5%: 45
(T−A)/T > 5%: 14
WNT7B chr22 45972580
P = 0.051
HR = 5.8
95% CI: 2–17
(T−A)/T < 5%: 12
(T−A)/T > 5%: 47
FZD10 chr12 130162026
P = 0.22
HR = 0.54
95% CI: 0.16–1.8
(T−A)/T < 6%: 13
(T−A)/T > 6%: 46
FZD10 chr12 130162056
P = 0.082
HR = 0.39
95% CI: 0.084–1.8
(T−A)/T < 6%: 8
(T−A)/T > 6%: 51
NOTCH1 chr9 136511336
P = 0.056
HR = 0.18
95% CI: 0.062–0.52
(T−A)/T < 6%: 47
(T−A)/T > 6%: 12
NOTCH1 chr9 136511366
P = 0.12
HR = 0.23
95% CI: 0.073–0.73
(T−A)/T < 6%: 47
(T−A)/T > 6%: 12
NOTCH1 chr9 136511379
P = 0.53
HR = 0.63
95% CI: 0.18–2.2
(T−A)/T < 6%: 49
(T−A)/T > 6%: 10
WNT7B chr22 45972580
P = 0.25
HR = 2
95% CI: 0.72–5.5
(T−A)/T < 6%: 15
(T−A)/T > 6%: 44
FZD10 chr12 130162026
P = 0.34
HR = 0.51
95% CI: 0.19–2
(T−A)/T < 7%: 14
(T−A)/T > 7%: 45
FZD10 chr12 130162056
P = 0.082
HR = 0.39
95% CI: 0.084–1.8
(T−A)/T < 7%: 8
(T−A)/T > 7%: 51
NOTCH1 chr9 136511336
P = 0.12
HR = 0.23
95% CI: 0.073–0.73
(T−A)/T < 7%: 49
(T−A)/T > 7%: 10
NOTCH1 chr9 136511366
P = 0.27
HR = 0.34
95% CI: 0.092–1.3
(T−A)/T < 7%: 50
(T−A)/T > 7%: 9
NOTCH1 chr9 136511379
P = 0.28
HR = 0.35
95% CI: 0.094–1.3
(T−A)/T < 7%: 52
(T−A)/T > 7%: 7
WNT7B chr22 45972580
P = 0.12
HR = 2.6
95% CI: 0.98–6.9
(T−A)/T < 7%: 17
(T−A)/T > 7%: 42
FZD10 chr12 130162026
P = 0.34
HR = 0.61
95% CI: 0.19–2
(T−A)/T < 8%: 14
(T−A)/T > 8%: 45
FZD10 chr12 130162056
P = 0.14
HR = 0.45
95% CI: 0.11–1.9
(T−A)/T < 8%: 9
(T−A)/T > 8%: 50
NOTCH1 chr9 136511336
P = 0.048
HR = 0
95% CI: 0–0
(T−A)/T < 8%: 51
(T−A)/T > 8%: 8
NOTCH1 chr9 136511366
P = 0.5
HR = 0.5
95% CI: 0.11–2.3
(T−A)/T < 8%: 53
(T−A)/T > 8%: 6
NOTCH1 chr9 136511379
P = 0.47
HR = 0.48
95% CI: 0.11–2.1
(T−A)/T < 8%: 54
(T−A)/T > 8%: 5
WNT7B chr22 45972580
P = 0.82
HR = 1.1
95% CI: 0.42–2.9
(T−A)/T < 8%: 20
(T−A)/T > 8%: 39
FZD10 chr12 130162026
P = 0.43
HR = 0.67
95% CI: 0.21–2.1
(T−A)/T < 9%: 15
(T−A)/T > 9%: 44
FZD10 chr12 130162056
P = 0.21
HR = 0.51
95% CI: 0.13–2.1
(T−A)/T < 9%: 10
(T−A)/T > 9%: 49
NOTCH1 chr9 136511336
P = 0.048
HR = 0
95% CI: 0–0
(T−A)/T < 9%: 51
(T−A)/T > 9%: 8
NOTCH1 chr9 136511366
P = 0.59
HR = 0.58
95% CI: 0.12–2.9
(T−A)/T < 9%: 54
(T−A)/T > 9%: 5
NOTCH1 chr9 136511379
Insufficient group sizes
WNT7B chr22 45972580
P = 0.63
HR = 1.3
95% CI: 0.5–3.4
(T−A)/T < 9%: 22
(T−A)/T > 9%: 37
FZD10 chr12 130162026
P = 0.58
HR = 0.75
95% CI: 0.25–2.3
(T−A)/T < 10%: 16
(T−A)/T > 10%: 43
FZD10 chr12 130162056
P = 0.56
HR = 0.72
95% CI: 0.21–2.5
(T−A)/T < 10%: 12
(T−A)/T > 10%: 47
NOTCH1 chr9 136511336
P = 0.2
HR = 0
95% CI: 0–0
(T−A)/T < 10%: 55
(T−A)/T > 10%: 4
NOTCH1 chr9 136511366
P = 0.71
HR = 0.68
95% CI: 0.12–3.8
(T−A)/T < 10%: 55
(T−A)/T > 10%: 4
NOTCH1 chr9 136511379
Insufficient group sizes
WNT7B chr22 45972580
P = 0.88
HR = 1.1
95% CI: 0.44–2.8
(T−A)/T < 10%: 27
(T−A)/T > 10%: 32
Proportion surviving
Time (days)

Figure EV5. Kaplan–Meier survival curves illustrating the relationship between shallow DNA methylation changes at individual CpG sites and overall survival, using 3% to 10% methylation difference thresholds.

P values of K–M survival analysis were calculated using chi-squared distribution.

 © The Author(s)
